# Enamel matrix acidification caused by loss of amelogenin phosphorylation disrupts ameloblast pH-regulatory machinery

**DOI:** 10.1093/jbmrpl/ziag096

**Published:** 2026-06-01

**Authors:** Ai Thu Bui, Lasya Bhogadi, Henry C Margolis, Elia Beniash

**Affiliations:** Department of Oral and Craniofacial Sciences, University of Pittsburgh School of Dental Medicine (UPSDM), Pittsburgh, PA 15261, United States; Center for Craniofacial Regeneration, Pittsburgh, PA 15261, United States; Department of Oral and Craniofacial Sciences, University of Pittsburgh School of Dental Medicine (UPSDM), Pittsburgh, PA 15261, United States; Center for Craniofacial Regeneration, Pittsburgh, PA 15261, United States; Department of Oral and Craniofacial Sciences, University of Pittsburgh School of Dental Medicine (UPSDM), Pittsburgh, PA 15261, United States; Center for Craniofacial Regeneration, Pittsburgh, PA 15261, United States; Department of Periodontics and Preventive Dentistry, UPSDM, Pittsburgh, PA 15261, United States; Department of Oral and Craniofacial Sciences, University of Pittsburgh School of Dental Medicine (UPSDM), Pittsburgh, PA 15261, United States; Center for Craniofacial Regeneration, Pittsburgh, PA 15261, United States

**Keywords:** biomineralization, pH regulation, protein phosphorylation, immunochemistry, PCR, western blot, amelogenesis, carbonic anhydrases, ion transporters, microdissection

## Abstract

Amelogenin (AMELX), the predominant extracellular enamel matrix protein, contains a single phosphorylation site at serine16 (S16). To investigate the functional significance of AMELX phosphorylation, we generated a knock-in (KI) mouse model (*Amelx^S16A^*) in which S16 is substituted with alanine, preventing AMELX phosphorylation. These KI mice exhibited hypoplastic, hypomineralized enamel lacking a characteristic rod structure, demonstrating that AMELX phosphorylation is critical for dental enamel formation. Mineralization analysis revealed accelerated formation of carbonated hydroxyapatite (CHA) and rapid transformation of amorphous calcium phosphate to CHA during the secretory stage, resulting in enamel acidification. Based on these findings, we hypothesized that excessive acidity disrupts pH regulation in KI ameloblasts. To test this hypothesis, we examined the mRNA and protein expression of 3 carbonic anhydrases (CAs) involved in pH regulation: intracellular CA2, transmembrane CA9, and extracellular CA6, localized their distribution; and measured total CA enzymatic activity in enamel organs (EOs) and matrix lysates. Additionally, we assessed the mRNA expression of 4 ion transporters (Slc4a4, Slc24a3, Slc24a4, and Cftr) involved in pH regulation. Our results revealed significant downregulation of all studied genes during the secretory stage in KI vs WT mice, with a general trend of reduced expression during maturation. Consistent with these findings, CA activity was markedly lower in KI EOs. Collectively, these data indicate that AMELX phosphorylation supports ameloblast function by stabilizing enamel pH during the secretory stage of amelogenesis through its capacity to slow the rate of enamel mineral formation and prevent enamel acidification. In the absence of AMELX phosphorylation, excessive acidity overwhelms ameloblast pH regulation, leading to dysregulation and defective enamel. This study provides evidence that the loss of biological control over extracellular mineralization can lead to cellular disruption, highlighting a critical link between protein phosphorylation, mineralization kinetics, and pH regulation.

## Introduction

Mature dental enamel is an acellular tissue comprising the outermost layer of the tooth crown. It is the most highly mineralized tissue in mammals, comprised of up to 96% of a carbonated hydroxyapatite (CHA)-like mineral by weight. The primary function of the enamel layer is mastication, and it uniquely combines high hardness with fracture toughness, allowing it to endure a lifetime of use without catastrophic failure, despite its inability to self-repair. This exceptional mechanical resilience stems from the hierarchical structure of mammalian enamel.^1–4^ Its basic building block, the enamel rod, consists of parallel arrays of elongated mineral crystals that are roughly 50 nm wide and tens of microns long,^5^ arranged into an intricate decussating pattern,^3^^,^^4^ which toughens enamel by arresting crack propagation.^1^^,^^2^ Remaining water and residual enamel matrix proteins (EMPs) also contribute to the mechanical properties of enamel.^1^^,^^2^

This elaborate structure is formed by highly specialized epithelial cells, called ameloblasts, through a multi-step process of amelogenesis involving distinct cellular phenotypes (Figure S1).^6^^,^^7^ The process begins when inner enamel epithelium cells differentiate into preameloblasts, and then into presecretory ameloblasts, which degrade the basal lamina and secrete a thin, rodless enamel layer on the top surface of dentin. The initial enamel contains approximately one-third mineral, in the form of 5 nm thick and 20 nm wide ribbons, one-third EMPs and one-third water. The first mineral phase in forming enamel is amorphous calcium phosphate (ACP), which subsequently transforms into non-stoichiometric CHA crystals.^8–10^ The enamel matrix (EM) consists primarily of amelogenin (AMELX), accounting for 90% of its total protein content, with ameloblastin, and enamelin and matrix metalloproteinase-20 comprising the rest.^6^ Each secretory-stage ameloblast develops a highly specialized secretory apparatus called a Tomes’ process, which produces 1 enamel rod.^6^ As illustrated in Figures S1 and S2, ameloblasts move away from the dentino-enamel interface in groups along predetermined trajectories, leaving behind the secreted enamel rods to form an intricate decussating pattern of prismatic enamel. Upon reaching full enamel thickness, ameloblasts transition by resorbing their Tomes’ processes to form a rodless outer enamel layer, on top of which a new basal lamina is then formed. This short-lived stage, called the transition stage, is followed by the maturation stage.^6^ During the maturation stage, the full densification of the enamel layer is achieved by the removal of EMPs^11^^,^^12^ and the overgrowth of the preexisting crystallites, which serve as a template for the mature enamel structure.^5^

Throughout amelogenesis, the pH of the enamel space is under tight biological control. During the secretory stage, the pH is strictly maintained near neutrality, whereas in the maturation stage, it fluctuates between neutrality and mild acidity,^12^ as ameloblasts undergo several modulation cycles between ruffle-ended and smooth-ended phenotypes (Figure S1) until the enamel reaches full maturity.^7^ Enamel pH is acidic beneath ruffle-ended ameloblasts and neutral beneath smooth-ended ameloblasts.^7^^,^^13^ This tight control of enamel pH during amelogenesis is carried out by an elaborate regulatory machinery, including carbonic anhydrases (CAs) and ion transporters.^6^

Carbonic anhydrases are a large enzyme family that modulates the carbonate-bicarbonate balance, and several isoforms, including CA2, CA6, and CA9, are strongly expressed by ameloblasts during enamel formation.^14^^,^^15^ CA2 is a cytoplasmic CA that plays a critical role in regulating intracellular pH and facilitating transmembrane bicarbonate flux by forming complexes with ion transporters and modulating their activity.^16^ CA2 is expressed throughout amelogenesis; however, its expression is higher during the maturation stage, where it is localized at the distal ends of ameloblasts.^14^^,^^15^^,^^17^^,^^18^ Mutations in the CA2 gene cause CA2 deficiency syndrome, characterized by renal tubular acidosis, cerebral calcifications, osteopetrosis, and severe syndromic amelogenesis imperfecta (AI).^19^^,^^20^ CA9 is a transmembrane isoform with an extracellular catalytic domain and a short cytoplasmic tail. It is primarily expressed in gastric tissues and solid tumors, where it protects cells from acidic environments, often through interactions with ion transporters.^16^ In the enamel organ (EO), CA9 is expressed throughout amelogenesis, with the strongest signal in maturation-stage cells.^15^ CA6 is a secreted isoform abundant in saliva. Initially identified as “gustin” for its role in taste bud development, it exists in both secreted (type A) and intracellular (type B) forms.^21^^,^^22^ CA6 expression in the EO has been confirmed during the secretory and maturation stages by RT-qPCR and immunohistochemistry (IHC), with the strongest expression in transition- and maturation-stage ameloblasts.^14^^,^^15^^,^^23^

Together, these CAs contribute to pH regulation by catalyzing the reversible hydration of CO₂, thereby influencing bicarbonate availability. However, effective pH homeostasis during enamel formation also relies on the coordinated activity of ion transporters, which facilitate the movement of bicarbonate, protons, and other ions across cellular membranes. SLC4A4 encodes the Na^+^/HCO₃^−^ co-transporter NBCe1, which is essential for maintaining pH balance in the EO.^24^ Mutations in SLC4A4 cause syndromic AI, proximal tubular acidosis, and glaucoma.^25^ NBCe1 shows dynamic expression in the EO: weak in presecretory and secretory ameloblasts, increasing during transition, disappearing briefly, and peaking again in mid-maturation.^24^ It localizes to basolateral membranes and Tomes’ processes.^24^^,^^26^ SLC24A4 encodes the Na^+^/K^+^/Ca^2+^ exchanger NCKX4, which is strongly expressed in maturation-stage ameloblasts and localized to their apical membranes.^27–29^ Mutations in SLC24A4 result in enamel maturation defects. SLC24A3, another member of the same family, encodes NCKX3 and is expressed in both secretory and maturation-stage ameloblasts, with higher levels during the secretory stage; it is most abundant in the Tomes’ processes of secretory ameloblasts.^27^ Cystic Fibrosis Transmembrane Conductance Regulator (CFTR), encoded by Cftr, is a Cl^−^/HCO₃^−^ transporter active during the maturation stage, where it helps neutralize protons released during mineralization by transporting Cl^−^ into the enamel space.^30^^,^^31^ Enamel defects are commonly observed in cystic fibrosis patients.^32^

Within this tightly regulated environment, EMPs play critical roles in coordinating mineral deposition. Amelogenin, the most abundant EMP, contains highly conserved N- and C-terminal domains and a more variable, Pro- and Gln-rich central domain.^33^ The AMELX gene is located on the X-chromosome and its mutations cause X-linked AI.^34^ Amelogenin undergoes a single posttranslational modification—the phosphorylation at serine16 (S16)^35^ within the highly conserved N-terminal domain.^36^ Using knock out (KO) mouse models, AMELX has been shown to play a critical role in regulating enamel mineral formation.^37^^,^^38^ In vitro, full-length AMELX has been shown to regulate the organization of mineral crystallites into parallel arrays resembling the mineral organization in enamel rods.^39–42^ Full-length AMELX undergoes hierarchical assembly, and the nascent enamel structure emerges through the cooperative interactions between forming crystals and assembling proteins.^39^

In vitro studies from our laboratory^40^^,^^43^^,^^44^ have demonstrated that phosphorylated AMELX stabilizes ACP and inhibits apatite formation. To assess the role of AMELX phosphorylation in vivo we generated a knock-in (KI) mouse model in which S16 is substituted with Ala (*Amelx*^S16A^) thereby preventing AMELX phosphorylation.^9^ Knock-in enamel exhibits a dramatic phenotype: it is hypoplastic, hypomineralized, lacks enamel rods and presents with numerous ectopic calcifications.^9^^,^^45^^,^^46^ Similar phenotypes with hypoplastic rodless enamel were observed in mice lacking a N-terminal fragment of AMELX containing S16,^47^ mice with defects in the major Golgi kinase Fam20C and its chaperone, pseudokinase Fam20A,^48^ and in Amelx KO mice.^37^ These findings demonstrate that phosphorylated AMELX is essential for proper enamel formation. Notably, our studies revealed that the absence of AMELX phosphorylation disrupts the biological control of enamel mineralization, accelerating the transformation of ACP to CHA and increasing mineral deposition.^9^^,^^45^^,^^46^ Accelerated deposition of CHA during the secretory stage of KI enamel formation leads to the release of excess protons, resulting in enamel acidification.^9^^,^^46^ This discovery led us to hypothesize that the pH regulatory mechanisms in the KI mice are compromised by the resulting acidic environment.

To test this hypothesis, we examined the expression and localization of key pH regulatory components in EOs of mandibular incisors from 12-wk-old WT and *Amelx^S16A^* KI mice. Specifically, we analyzed the mRNA levels, protein abundance, and histological distribution of 3 CA isoforms—cytoplasmic CA2, transmembrane CA9, and extracellular CA6, which are highly expressed in ameloblasts,^14^ in the EOs of mandibular incisors of 12-wk-old (wo) WT and KI mice. In addition, we assessed the mRNA expression of 4 other pH regulators known to be active in ameloblasts: *Slc4a4, Slc24a4, Slc24a3, and Cftr.*^6^ These analyses aimed to determine whether the acidic environment caused by uncontrolled mineralization in the absence of phosphorylated AMELX in KI enamel disrupts the normal function of pH regulatory machinery, thereby contributing to the pathological mineralization phenotype.

**Figure 1:**
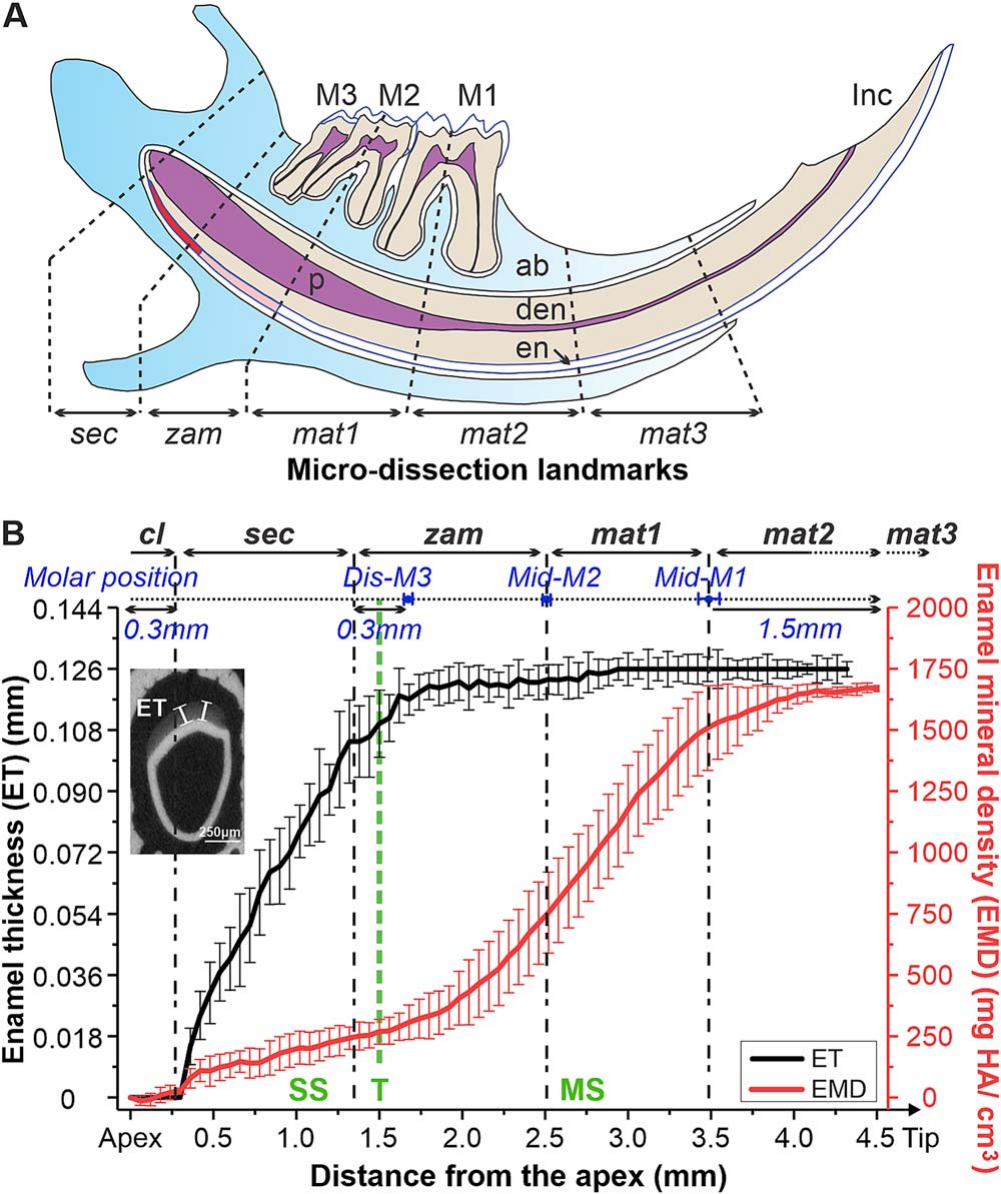
(A) Scheme, illustrating the strip microdissection of the mandibular incisor enamel organs (EOs) using molar landmarks. The scheme presents landmarks used for micro-dissecting the EO epithelium (EOE) layer of mandible incisors. The EOE is divided into 5 different segments, *sec*, *zam*, *mat1*, *mat2*, and *mat3*, containing ameloblasts of different stages of amelogenesis; M1, M2, M3, first, second, and third molars; Inc, incisors; en, enamel; den, dentin; p, pulp; ab, alveolar bone. (B) Graph showing the enamel thickness (ET; black solid line) and enamel mineral density (EMD; red solid line) in mouse incisors with respect to the stages of amelogenesis, based on micro-CT data (reanalyzed from^49^). Scanning data from 4 independent male WT incisors are reorientated (following approach 2, described in^46^) with the region of interest located at the first 4.5 mm starting from the apex of the incisors (the onset of the dentin). The ET (in mm) and EMD (in mg HA/ cm^3^) profiles are shown at each 60 μm paces from the incisor apex, graphed as mean ± SD. Blue text shows molar positions. Black dash dot lines show the dissection landmarks. The first dissecting line is to remove 0.3 mm EOE tissue at the cervical loop containing undifferentiated epithelium and contaminated mesenchyme. The second line locates at 0.3 mm distally from the distal end of the M3 (dis-M3). The third line and the fourth line locate at middle of M2 (mid-M2) and M1 (mid-M1), respectively. The fifth line is 1.5 mm mesial to the Mid-M1. Bold green line shows the location of the transition stage of ameloblasts (adapted from^49^). Green text SS, T, and MS represent secretory stage, transition, and maturation stage, corresponding to the morphology of ameloblasts through amelogenesis.

## Materials and methods

A detailed description of materials and methods is in Supplementary material file.

### Animals and sample collection

Amelx^S16A^ KI (KI) mice on a C57BL/6 background were generated as previously described^9^; they were maintained and euthanized at the University of Pittsburgh Division of Laboratory Animal Resources facility under approved protocols. Five-day-old (-do) to 9-do and 12-wo WT, HET and KI mice were used in this study.

### Microdissection, RNA, and protein extraction from mandibular incisors and molars

We utilized a strip microdissection technique recently developed in our laboratory^49^ (Figure 1A) to perform high-resolution analyses of mRNA expression and protein distribution across different stages of enamel development in the WT and Amelx^S16A^ KI mandibular incisal EOs. The structure of the mandibular incisal EO and the microdissection scheme utilized are illustrated in Figure 1A, with abbreviations and strip nomenclature defined in the legend. This method employs molar teeth as anatomical landmarks to delineate specific stages of amelogenesis, as previously characterized in the WT mice.^49^ as shown in Figure 1A. This approach enabled us to spatially map changes in the mRNA and protein levels of CAs and other pH regulators throughout amelogenesis and correlate these molecular changes with enamel layer thickening and mineral densification (Figure 1B). It is important to note that the dissected strips used for RT-qPCR and western blot (WB) analyses included all EO tissues—stratum intermedium (SI), papillary layer (PL), blood capillaries, and outer enamel epithelium—not just ameloblasts. Therefore, the expression profiles reported reflect total EO expression at each stage of enamel development. To complement these molecular analyses, we performed a quantitative image analysis of IHC micrographs to assess the relative signal intensity and spatial distribution of proteins within specific EO cell layers and structures.

Immunohistochemistry and image analysis were conducted using protocols developed in our lab,^50^ as described in Supplementary material.

### Determination of Ca activity of EOs and matrices using a nitrophenol assay

Secretory-stage incisal EOs and EMs were collected, lysed in RIPA buffer, and assayed using a Ca activity kit (Abcam), following a standard protocol^51^ and the manufacturer’s instructions. The outcomes of these assays provide standardized assessments of site-specific Ca activities.

### Statistical analysis

Statistical analyses were carried out using ANOVA with post hoc multiple comparisons tests*,* as described in Supplementary material.

## Results

### Statistical analysis of the data

The mRNA expression data for CA2, CA6, and CA9 (Tables S3, S6, and S9) were first analyzed using a 3-way ANOVA test, with sex, segment, and genotype as factors. Since sex was not a significant variable, data from both sexes were pooled and reanalyzed using a 2-way ANOVA with segment and genotype as factors. Significant differences between individual means were identified using Tukey’s multiple comparison test (Tables S3, S6, and  S9). These analyses showed that heterozygous (HET) mice generally exhibited intermediate expression levels between the WT and KI groups, without a consistent trend. For clarity, HET data will not be discussed further.

We also performed a 2-way ANOVA on the WB data for CA2 and CA9 protein levels, using genotype and segment as factors. For CA2, genotype was a significant factor, while segment was not (Table S4). In contrast, both genotype and segment significantly affected CA9 levels (Table S7). For CA6 protein levels in the EM, we evaluated age (5-8 d) and genotype as factors and found that only genotype had a significant effect. A summary of the RT-qPCR and WB results is presented in Tables 1 and 2.

**Table 1:** Summary of mRNA expression data.

**Gene name**	**mRNA expression in the 5 segments of the incisal enamel organ**									
	**WT**					**Amelx** ^**S16A**^ **KI**				
	***sec***	***zam***	***mat1***	***mat2***	***mat3***	***sec***	***zam***	***mat1***	***mat2***	***mat3***
***Ca2***	***	****	****	****	****	**↘	****	****	****	****
***Ca9***	**	*	*	*	*	**↘	**↗	*	*	*
***Ca6***	***	****	*	*	*	*↘	***↘	*	*	*
***Slc4a4***	**	**	**	*	*	*↘	**	**	*	*
***Slc24a4***	*****	*******	******	****	**	*↘	*****↘	******	****↗	**
***Slc24a3***	***	**	*	*	*	***	***↗	*	*	*
***Cftr***	****	*****	*****	*****	*****	*↘	****↘	****↘	****↘	****↘

Asterisks indicate the strength of expression. Arrows show expression increase or decrease in Amelx^S16A^ KI mice.

**Table 2:** Summary of protein abundance data.

**Gene name**	**Protein abundance in the 5 segments of the incisal enamel organ**									
	**WT**					**Amelx** ^**S16A**^ **KI**				
	***sec***	***zam***	***mat1***	***mat2***	***mat3***	***sec***	***zam***	***mat1***	***mat2***	***mat3***
**Ca2**	***	***	**	**	***	**↘	***	****↗	****↗	****↗
**Ca9**	***	****	****	****	***	**↘	***↘	***↘	***↘	**↘
	Protein abundance in the first molar enamel matrix at different ages									
	5-do	6-do	7-do	8-do	9-do	5-do	6-do	7-do	8-do	9-do
**Ca6**	***	***	***	**	**	*↘	*↘	*↘	*↘	*↘

Asterisks indicate the strength of expression. Arrows show expression increase or decrease in Amelx^S16A^ KI mice. For Ca6, d 5 and d 6 correspond to secretory, d 7 to transition and early maturation, and d 8 and d 9 to mid-maturation stages.

### CA2 mRNA expression, protein abundance and distribution in the EOs of mandibular incisors of WT and Amelx^S16A^ KI mice

**Figure 2:**
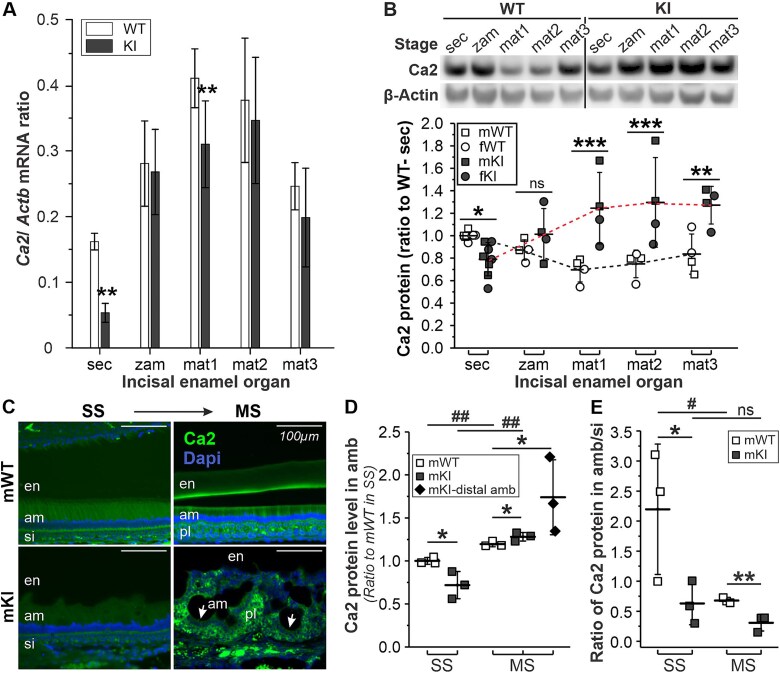
mRNA expression, protein abundance, and distribution of Ca2 in enamel organs (EOs) of mandibular incisors of WT and *Amelx^S16A^* KI mice. (A) mRNA expression in the 5 segments of the EOs, as outlined in Figure 1, normalized to the β-actin expression. (B) Representative cutouts of western blots from the 5 segments of EOs stained against Ca2 and β-actin (upper row) and relative protein abundance calculated using optical density (OD) values of the WB bands of Ca2 protein in the same 5 segments normalized to β-actin (*n* = 4, 2 males, 2 females). WT *sec* OD value is set as 1. (C) Representative immunofluorescence micrographs expression level and distribution of Ca2 of secretory and maturation stage EOs in WT and Amelx^S16A^ KI males (scale bars: 100 μm). (D) Quantitative image analysis of the fluorescence signal intensity in WT and Amelx^S16A^ KI calculated from the mean gray values (MGVs) secretory and maturation stage ameloblasts expressed as ratios to MGV of WT secretory stage ameloblasts. (E) Ratios of the fluorescence signal intensity (MGVs) in WT and *Amelx^S16A^* KI ameloblasts vs SI or PL. am: ameloblast; en: enamel; si: stratum intermedium; pl: papillary layer. ^*^Difference between KI and WT; ^*^p ≤ .05, ^**^p ≤ .01, ^***^p ≤ .001. ^#^: difference between level of expression in secretory and maturation stages in the same genotype; ^#^*p* ≤ .05, ^##^*p* ≤ .01, ^###^*p* ≤ .001.

In the WT mice, CA2 mRNA expression was lowest in the sec segment and progressively increased through the *zam* and *mat1* segments, plateaued in *mat2*, and then declined to sec levels in *mat3* (Figure 2A). However, WB analysis did not reflect this trend; instead, CA2 protein abundance was highest in the sec segment and remained at 60%-80% of that level across the *zam* to *mat3* segments (Figure 2B).

Immunofluorescence (IF) IHC revealed the CA2 signal in secretory (SA) and maturation (MA) ameloblasts, the SI, and PL cells in the WT mice (Figure 2C). In SA, the CA2 signal was present throughout the cell body, while in MA the signal was concentrated at the distal ends of ameloblasts (Figure 2C and D), consistent with previous reports.^15^^,^^17^ Quantitative image analysis showed a moderate but statistically significant (~20%) increase in Ca2 signal from the secretory to maturation stage (Figure 2D), in agreement with literature findings.^15^^,^^18^ The signal in the SI layer, however, was ~2.2 times weaker than in the underlying SA, whereas during maturation, the PL signal was ~1.5 times stronger than in MA (Figure 2E).

These findings confirm that CA2 expression in ameloblasts increases during the maturation stage in the WT mice, aligning with previous findings.^17^ Additionally, our data support earlier observations that CA2 localizes to the distal regions of ameloblasts during maturation.^15^^,^^18^

In the KI mice, CA2 mRNA expression in the *sec* segment was significantly lower than in the WT (Figure 2A; Table 1; Table S3), though the overall expression pattern across segments mirrored that of the WT. Western blot analysis corroborated the reduced protein abundance in the KI sec segment, showing ~30% lower levels compared to the WT (Figure 2B; Table 2). While protein levels in the *zam* segment were similar between genotypes, CA2 abundance was significantly higher in the KI mice across mat1-mat3, with the most pronounced increase (1.8-fold) in mat1 (Figure 2B), contrasting with the corresponding mRNA data.

Furthermore, IF analysis revealed that the CA2 signal in KI secretory ameloblasts was ~1.4 times lower than in the WT (Figure 2C and D). During maturation, in KI ameloblasts, the overall signal intensity was 7% higher in MA then in SA, with a pronounced signal concentration at the distal ends. When mean gray values (MGVs) were measured specifically in the distal regions, the CA2 signal in KI was 1.5 times greater than in the WT (Figure 2D). Unlike WT, KI mice showed marked differences in the CA2 signal between ameloblasts and adjacent SI/PL layers. Notably, during maturation, the CA2 signal in ameloblasts was 3.3 times lower than in PL cells (Figure 2E). These findings may explain the elevated CA2 protein levels observed in KI mat1-mat3 segments vs WB (Figure 2B).

Overall, these results demonstrate that the absence of AMELX phosphorylation alters CA2 mRNA expression, protein abundance, and spatial distribution. A consistent reduction in CA2 expression during the secretory stage was observed across all analytical methods used (Tables 1 and 2). However, the maturation stage presents a more complex picture, with discrepancies between mRNA and protein levels. In the WT mice, mRNA expression increases from zam to mat3, while protein levels decline. In the KI mice, despite lower or unchanged mRNA levels, CA2 protein abundance increases. Such divergence between transcription and protein levels is well-documented and may be attributed to differences in molecular half-life and translation kinetics.^52^^,^^53^ Collectively, our findings demonstrate a reduced CA2 expression in KI ameloblasts, particularly during the secretory stage, which may impair their ability to regulate extracellular pH effectively.

### CA9 mRNA expression, protein abundance, and distribution in the EOs of mandibular incisors of WT and Amelx^S16A^ KI mice

**Figure 3:**
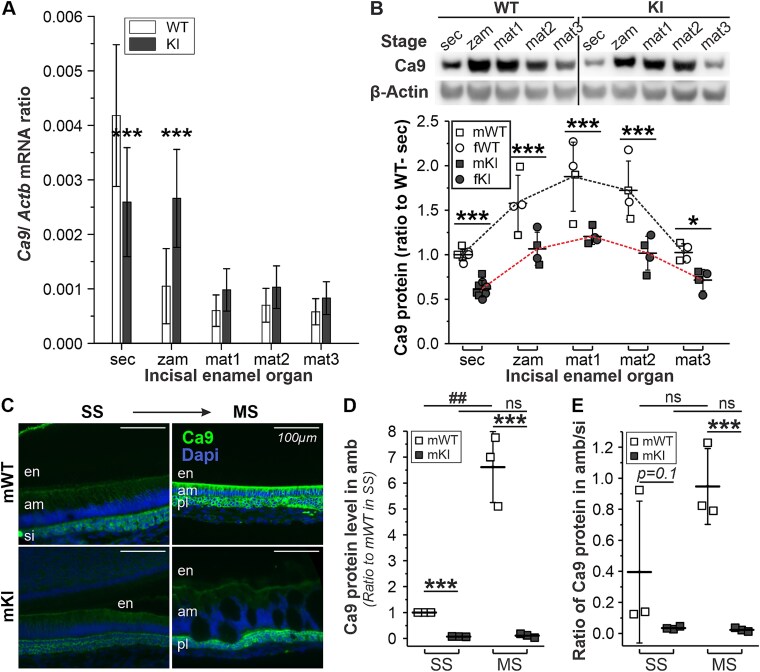
mRNA expression, protein abundance, and distribution of Ca9 in enamel organs (EOs) of mandibular incisors of WT and *Amelx^S16A^* KI mice. (A) mRNA expression in the 5 segments of the EOs, as outlined in Figure 1, normalized to the β-actin expression. (B) Representative cutouts of western blots from the 5 segments of EOs stained against Ca9 and β-actin (upper row) and relative protein abundance calculated using optical density (OD) values of the WB bands of Ca9 protein in the same 5 segments normalized to β-actin (*n* = 4, 2 males, 2 females). WT *sec* OD value is set as 1. (C) Representative immunofluorescence micrographs expression level and distribution of Ca9 of secretory and maturation stage EOs in WT and Amelx^S16A^ KI males (scale bars: 100 μm). (D) Quantitative image analysis of the fluorescence signal intensity in WT and Amelx^S16A^ KI calculated from the mean gray values (MGVs) secretory and maturation stage ameloblasts expressed as ratios to MGV of WT secretory stage ameloblasts. (E) Ratios of the fluorescence signal intensity (MGVs) in WT and Amelx^S16A^ KI ameloblasts vs SI or PL. am: ameloblast; en: enamel; si: stratum intermedium; pl: papillary layer. ^*^: Difference between KI and WT; ^*^p ≤ .05, ^**^p ≤ .01, ^***^p ≤ .001. #: difference between level of expression in secretory and maturation stages in the same genotype; #*p* ≤ .05, ^##^*p* ≤ .01, ^###^*p* ≤ .001.

In the WT EOs, CA9 mRNA expression was highest in the *sec* segment, followed by a sharp 4-fold decline in the *zam* segment, remaining low throughout the maturation stages (Figure 3A; Table S6). This observation contrasts with CA2, whose expression increases during maturation (Figure 2A). Interestingly, CA9 protein abundance in the WT mice did not mirror its mRNA profile. While mRNA levels were highest in *sec* and nearly undetectable in the *zam-mat3* segments, protein levels were lowest in *sec* and gradually increased through *zam-mat2*, before returning to sec levels in *mat3* (Figure 3B).

Consistent with the WB data, immunohistochemical analysis in the WT mice revealed that the CA9 signal intensity in ameloblasts was 6.6 times higher during the maturation stage compared to the secretory stage. In MA, the signal was concentrated at the distal ends of the cells, whereas in SA, a weaker signal was distributed throughout the cell body (Figure 3C and D; Figure S5). Additionally, the CA9 signals in the SI and PL was 2.5 times stronger than in SA and MA, respectively (Figure 3D and F). These findings are consistent with previous reports showing high CA9 levels in maturation-stage ameloblasts, where the protein is localized to the distal portions of the cells.^15^

In the KI mice, CA9 mRNA expression followed a similar pattern to that of the WT, with the highest levels in the sec segment and the lowest in mat1-mat3. However, KI mice exhibited significantly higher CA9 mRNA expression in the zam segment compared to the WT, reaching levels comparable to those in sec (Figure 3A; Table 1). Despite this, CA9 protein levels in the KI mice were consistently lower than in the WT across all segments, although the overall profile was similar—lowest in sec, peaking in zam-mat2, and then declining in mat3 (Figure 3B; Table 2).

Comparative IF image analysis revealed a dramatic reduction in the CA9 signal in KI ameloblasts. Specifically, the CA9 signal was 14-fold lower in secretory-stage and 60-fold lower in maturation-stage ameloblasts compared to the WT (Figure 3C and D; Figure S5). In contrast, CA9 expression in SI and PL remained unchanged between genotypes (Figure 3C), which may explain why the WB profiles in the KI mirrored those of the WT despite lower overall protein levels.

Together, these results clearly demonstrate that the lack of AMELX phosphorylation affects the expression of CA9. Moreover, similar to CA2, the CA9 mRNA expression profile did not match changes in protein levels in both genotypes, potentially due to differences in the half-life of these macromolecules and/or their translation kinetics.^52^^,^^54^

Given CA9’s established role in protecting cells from acidosis,^16^ its elevated expression during the maturation stage in the WT mice may reflect a similar protective function during enamel formation. In the KI mice, reduced CA9 levels may compromise this defense, exacerbating the effects of enamel acidification due to the accelerated mineral deposition.^9^^,^^46^

### CA6 mRNA expression, protein abundance, and distribution in the EOs and enamel matrices of mandibular incisors and first molars of WT and AmelxS16A KI mice

**Figure 4:**
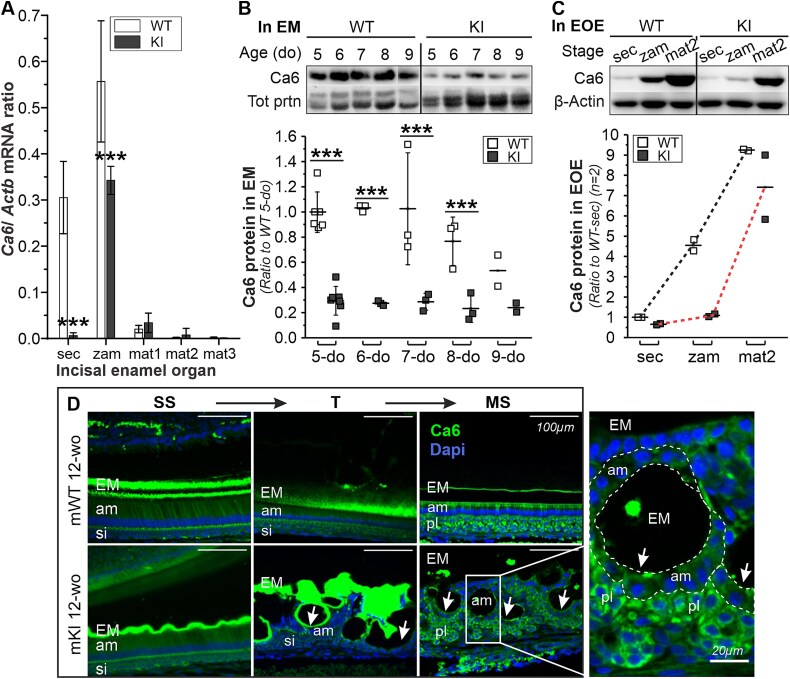
mRNA expression, protein abundance, and distribution of Ca6 in enamel organs (EOs) of mandibular incisors of WT and *Amelx^S16A^* KI mice. (A) Ca6 mRNA expression in the 5 segments of the EOs, as outlined in Figure 1, normalized to the β-actin expression. (B) Representative cutouts of WB bands from the enamel matrix from first molars of 5-do (*n* = 7), 6-do, 7-do, and 8-do (*n* = 3), and 9-do (*n* = 2) mice, stained against Ca6. Protein abundance is calculated using optical density (OD) values of the WB bands. Ca6 OD is normalized to total protein amount stained with loaded on SDS PAGE stained with SimplyBlue SafeStain. The OD of 5-do WT is set as 1. (C) Representative cutouts of WB bands cut from *sec*, *zam*, and *mat2* segments of EOs stained against Ca6 and β-actin and relative protein abundance calculated using OD values of the WB bands. Ca6 OD is normalized to β-actin and the OD of WT *sec* is set as 1. (D) Immunofluorescence images of secretory (SS), transition (T), and maturation (MS) stages of WT and Amelx^S16A^ KI incisal EOs (scale bars: 100 μm). The right inset highlights a spherulite (appearing as an empty space, noted as EM) and outlines the ameloblast layer (am) with dotted white line, indicated by arrows (scale bar: 20 μm). EM, enamel matrix; si: stratum intermedium; pl: papillary layer. ^*^: difference between genotypes; ^*^p ≤ .05, ^**^p ≤ .01, ^***^p ≤ .001.

The CA6 mRNA expression during amelogenesis was assessed by RT-qPCR in strip-dissected segments of EOs from 12-wk-ol mouse incisors (Figure 4A). Protein abundance was analyzed by WB in secretory-stage EM from first molars (M1) of 5-6 d-old pups, transition-stage EM from 7 d-old pups, and early maturation-stage EM from 8 d-old pups (Figure 4B).^55^ We also conducted WB on 9-d-old samples, but, because only 2 animals were collected at this time point, these data were excluded from the statistical analysis. In addition, we analyzed the protein abundance in the EO using strip-dissected segments of 12-wo mice (Figure 4C).

In the incisal EO, *CA6* mRNA expression levels were high during the active phases of the matrix deposition in the sec segment and attained their maximum in the zam segment, reaching ~56% of β-actin level (Figure 4A, Table S9). Expression dropped sharply—by 2 orders of magnitude—in the maturation segments (mat1-mat3) (Figure 4A). This pattern contrasts with earlier reports suggesting CA6 mRNA is expressed only during the maturation stage.^23^

WB analysis also revealed substantial CA6 protein levels in the secretory-stage EM from 5-d-old WT molars (Figure 4B). These levels remained high through the transition stage (6-7 d) and declined by ~50% by d 9, consistent with the proteolytic degradation of the matrix. In contrast, CA6 protein abundance increased in incisal EOs during maturation (Figure 4C), suggesting the presence of both extracellular (type A) and intracellular (type B) isoforms of CA6,^22^ expressed at different stages of amelogenesis.

IF IHC on mandibular incisors from 12-wo WT mice confirmed these findings. The CA6 signal was strong in the EM during the secretory and early maturation stages (Figure 4D). In secretory ameloblasts, the signal localized primarily to the Tomes’ processes, while in transition and early maturation stage ameloblasts, it was weaker. During maturation, the CA6 signal intensified in the apical regions of ameloblasts and the PL, consistent with previous reports.^15^ At the same time, the matrix signal diminished, reflecting matrix degradation. Overall, the IHC results aligned well with the WB data.

In the KI mice, CA6 mRNA expression was nearly absent in the sec segment but partially recovered in zam (Figure 4A). Expression in mat1-mat3 was barely detectable. Correspondingly, WB analysis showed that CA6 protein in the KI secretory EM (5-d-old molars) was ~3.4 times lower than in the WT and remained low throughout maturation (Figure 4B). In incisal EOs, Ca6 protein levels were low in the sec segment but increased 9-fold in zam and mat2 (Figure 4C), despite the absence of detectable mRNA in mat2 - suggesting the impact of post-transcriptional regulation or protein stability effects. In the KI, Ca6 protein levels remained low in sec and zam, followed by a sharp 7-fold increase in mat2. Due to limited sample availability (*n* = 2), statistical analysis was not performed for these data.

IHC of KI incisors revealed distinct differences in CA6 distribution (Figure 4D, lower panels). During the secretory stage, the Ca6 signal was concentrated at the apical ends of ameloblasts. A dramatic spike in the signal occurred in a narrow region spanning the transition and early maturation stages, followed by a near-complete loss during later maturation. This spike may correspond to the transient increase in mRNA expression observed in zam. CA6 remained associated with the apical boundary of ameloblasts and secretory aggregates throughout the EO epithelium.^9^^,^^45^ Similar to the WT, the CA6 signal in the KI diminished in the extracellular matrix during maturation, while expression in the PL increased. However, unlike the WT, the Ca6 signal in KI maturation-stage ameloblasts was barely detectable (Figure 4D, right inset).

These findings indicate that the absence of AMELX phosphorylation significantly alters Ca6 mRNA expression, protein abundance, and spatial distribution. CA6 is the only extracellular CA studied here, and it may play a critical role in pH regulation during enamel formation.

### CA activity in WT and Amelx^S16A^ KI enamel matrices and organs

**Figure 5:**
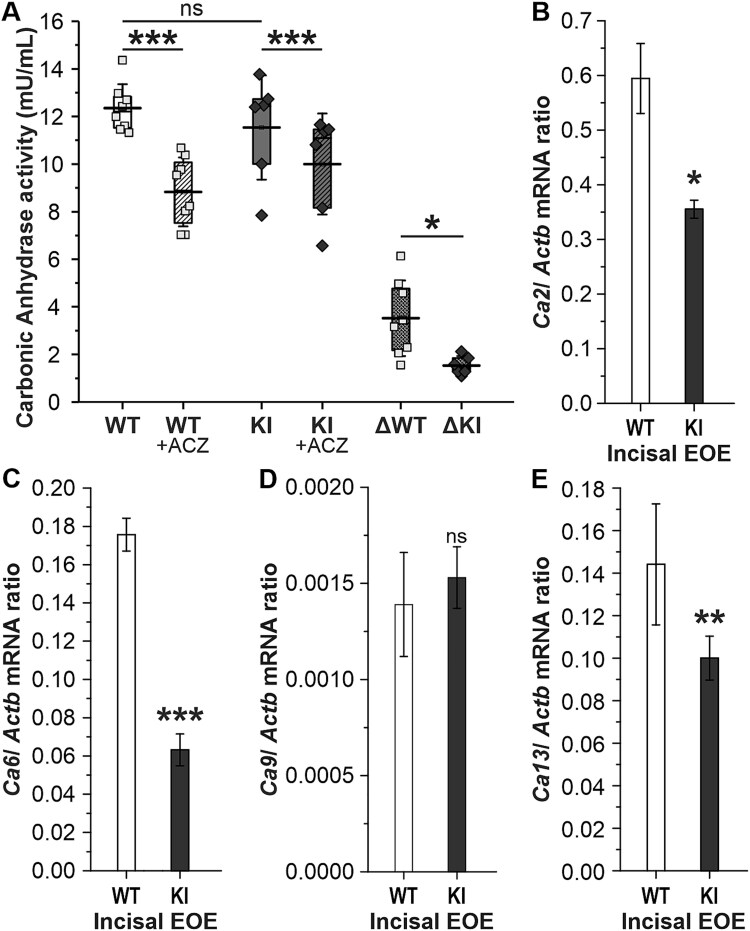
(A) Ca activity assay in the samples containing enamel organs (EOs) and enamel matrix from the mandibular incisors. +ACZ: with inhibitor acetazolamide; ΔKI and Δ WT are differences in activities of unadulterated samples and samples with ACZ; mRNA expression of *Ca2* (B); *Ca6* (C); *Ca9* (D); and *Ca13* (E) in total mandible EOs.

To assess functional Ca activity, esterase assays were performed on total protein extracts from whole EO layers and secretory EM of 12-wo WT and KI mouse incisors (Figure 5). To isolate specific Ca activity, assays were conducted with and without the Ca inhibitor acetazolamide (ACZ). Specific CA activities (Δ) were calculated by subtracting the esterase activity in the presence of ACZ and without ACZ.

In all samples, esterase activity was significantly reduced by ACZ (Figure 5A), confirming the presence of Ca activity. No sex-based differences were observed, so data from males and females were combined. Total CA activity in the KI samples was 2.3 times lower than in the WT, consistent with the reduced CA protein levels described above. These results align well with the mRNA and protein expression data from individual EO segments and total EO layers (Figure 5B-E).

### mRNA expression of ion transporters involved in pH regulation in mandibular incisors of WT and Amelx^S16A^ KI mice

**Figure 6:**
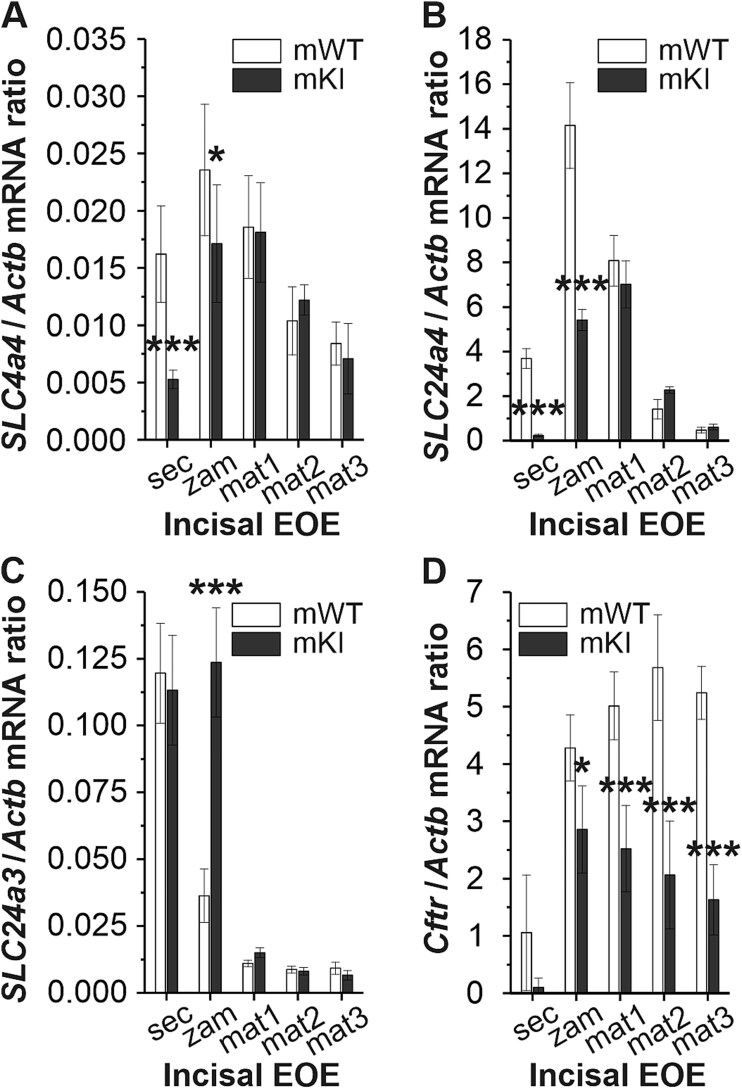
mRNA expression of pH regulators *Slc4a4* (A); *Slc24a4* (B); *Slc24a3* (D); and *Cftr* (E) in 5 segments of mandibular incisal enamel organs, as outlined in Figure 1, of WT and Amelx^S16A^ KI mice. The expression levels are normalized to the *β-actin* expression. ^*^: difference between genotypes; ^*^p ≤ .05, ^**^p ≤ .01, ^***^p ≤ .001.

In addition to the CA family, we analyzed the mRNA expression of 4 other pH regulators—*Slc4a4, Slc24a3, Slc24a4*, and *Cftr*—in incisal EOs of WT and KI mice.^6^ These genes are known to play critical roles in pH regulation during amelogenesis. In the WT mice, each gene exhibited a distinct expression profile across the stages of enamel development (Figure 6). Expression levels varied widely, ranging from <2% of β-actin for *Slc4a4* (Figure 6A) to up to 14-fold and 5.5-fold higher β-actin levels for *Slc24a4* and *Cftr*, respectively (Figure 6B and D).

In the WT EOs, *Slc4a4* mRNA expression was relatively strong in the sec segment, peaked in zam, and gradually declined through mat1–mat3 (Figure 6A). In the KI mice, expression was reduced by ~3-fold in sec and ~1.4-fold in zam compared to the WT, but remained unchanged in later segments, suggesting that AMELX phosphorylation primarily affects *Slc4a4* expression during the secretory stage.

In the WT, mRNA expression of *Slc24a4* was moderately high, roughly 4 times *β-actin* in *sec*; it peaked at ~14 times of β-actin in *zam* segment and declined through maturation (Figure 6B). This pattern aligns with previous reports, though our data show a smaller difference between secretory and maturation stages than the 3 orders of magnitude reported in the literature.^27^ At the same time, in our experiments the difference between secretory and maturation stages was <4 times, while Robertson et al.^27^ reported a difference of 3 orders of magnitude and much lower expression levels with respect to β-actin. In the KI mice, *Slc24a4* expression was ~16-fold lower in sec and ~2.6-fold lower in zam compared to the WT, with no difference in mat1, indicating that AMELX phosphorylation influences *Slc24a4* expression during early enamel mineralization.

In the WT, *Cftr* mRNA expression was substantially higher in *zam-mat3* segments than in *sec*, supporting the notion that it is active during the maturation stage of amelogenesis (Figure 6D).^31^ In KI, *Cftr* expression was lower in all segments when compared to the WT, suggesting that AMELX phosphorylation affects the ability of Cftr to regulate pH during amelogenesis. In the WT mice, *Cftr* mRNA expression was significantly higher in zam–mat3 segments than in sec, consistent with its role in maturation (Figure 6D). *Cftr* expression was reduced across all segments, indicating that AMELX phosphorylation may be necessary for proper CFTR-mediated pH regulation during amelogenesis.

## Discussion

The acidification of KI enamel caused by high rate of mineralization in the absence of phosphorylated Amelx during the secretory stage of amelogenesis^46^ indicates that in KI the pH regulatory machinery, normally maintaining enamel pH around neutrality,^7^^,^^12^ is affected. Three possible scenarios may explain this difference:

1) Genetically fixed pH regulation. The pH regulatory machinery may be genetically predetermined to counteract proton release associated with the low mineralization rates during the secretory stage and may be unresponsive to extracellular pH changes. As a result, excess acidity cannot be neutralized. In this scenario, the expression levels of the pH regulators would remain unchanged in KI.
2) Compensatory but insufficient response. The pH regulatory machinery may respond to acidification by increasing the production of the pH regulators; however, this response may be inadequate to fully neutralize the excess protons. In that case, expression of the pH regulatory genes would be elevated in KI ameloblasts.
3) Functional overload of the regulatory system. The degree of acidification may exceed the buffering and transport capacity of the pH regulatory machinery, resulting in functional overload. Under these conditions, the system becomes overwhelmed, and the expression of the pH regulatory genes would decline. The fact that the pH regulators are downregulated in the KI enamel favors this third scenario.

Here, the term “overwhelmed” refers to the physiological threshold at which the rate and magnitude of proton generation surpass the maximal capacity of ameloblast-mediated buffering and ion transport. Once this threshold is exceeded, pH can no longer be maintained near neutrality, leading to the dysregulation of pH regulatory gene expression. A similar phenomenon has been reported in the kidney under conditions of metabolic acidosis.^56^

Collectively, our results provide strong evidence that enamel acidification resulting from the absence of AMELX phosphorylation compromises the pH regulatory machinery during amelogenesis. This disruption is most pronounced during the secretory stage, when the rate of enamel mineralization is increased 3-fold in the KI enamel.^46^ At this stage, all examined pH regulators exhibited marked reductions in both protein abundance and mRNA expression in the KI ameloblasts (Tables 1 and 2), consistent with a global failure of the mechanisms that normally counterbalance the proton release associated with CHA formation.

Despite this overall downregulation, a subset of pH regulators displayed stage-specific deviations from this pattern. While most CAs and ion transporters showed reduced mRNA expression during early amelogenesis (Table 1), CA9 and Slc24a3 were notably upregulated during the transition/early maturation (zam) stage (Figures 3A and 6C; Table 1). In the WT mice, both genes are predominantly expressed during the secretory stage and are minimally expressed in later stages, suggesting that their coordinated upregulation in the KI enamel reflects an attempted, yet ultimately insufficient, compensatory response. The similarity in their expression profiles further suggests potential functional cooperation, possibly via the formation of a regulatory complex.^16^

The most plausible explanation for these findings is that the anomalously accelerated deposition of CHA in secretory-stage KI enamel,^46^ generates an excessive proton load that overwhelms the ameloblast pH regulatory system. Under normal conditions, enamel pH is tightly controlled through the coordinated activity of CAs, ion exchangers, and transporters, which balance acid production during mineralization.^7^^,^^12^^,^^57^^,^^58^ We have previously shown that phosphorylated AMELX plays an essential role in regulating the rate of mineral deposition during the secretory stage.^46^ Consistent with this framework, only ~17% (w/w) of enamel mineral is deposited during the secretory stage in the WT mice, with the majority occurring during maturation,^46^^,^^58^ when CAs and ion exchangers are more abundant and the pH regulatory demand is greater.^15^^,^^24^^,^^28^^,^^31^ The observed increase in the mineralization rate in KI enamel,^46^ however, leads to a commensurate increase in the proton concentration in the secretory enamel space, thereby increasing the acid load that must be neutralized by secretory-stage ameloblasts. This accelerated mineralization, coupled with reduced ameloblast pH regulatory activity in the absence of AMELX phosphorylation, as reported in the present study, results in enamel acidification,^46^ demonstrating that the KI regulatory machinery cannot accommodate the excessive proton load. In other words, the ameloblast buffering/transport system reaches its functional ceiling: even if compensatory pathways are present and engaged, their combined maximal capacity is insufficient to restore a near-neutral pH when proton production outpaces neutralization. Under such conditions, acidification itself may perturb pH-sensitive feedback mechanisms (including proton-sensing receptors and acid/base-coupled transporter regulation), thereby contributing to the observed dysregulation rather than eliciting expected adaptive upregulation.

Importantly, our data do not establish a direct causal signaling relationship in which AMELX phosphorylation transcriptionally regulates the expression of pH regulators. Rather, we interpret the altered expression levels, abundance, and spatial distribution of the pH regulators as a response to the altered extracellular physicochemical environment created by the accelerated mineralization during the secretory stage of enamel formation, as discussed above. At the same time, AMELX phosphorylation may exert additional effects that contribute to the KI ameloblast phenotype. We have recently demonstrated that the phosphorylation status of AMELX influences its interactions with other proteins,^59^ which in turn may alter ameloblast-matrix interactions and drive broader changes in ameloblast biology.

The enamel phenotypes observed in the KI mice parallel those reported in several human X-linked AI cases and genetic animal models. Patients with amorphic *AMELX* mutations that prevent protein secretion,^60^ and Amelx KO mice exhibit rodless, hypoplastic, and hypomineralized enamel with pitted enamel surfaces and ectopic calcifications.^38^ Importantly, in the Amelx KO, enamel exhibits the prominent presence of large, plate-like crystals of octacalcium phosphate,^38^ a mineral phase that more readily forms under acidic conditions,^61^ indicating a more acidic microenvironment during enamel mineralization, although enamel pH was not assessed in that study. Nevertheless, the similarity between these phenotypes and the Amelx^S16A^ KI, which lacks Amelx phosphorylation, emphasizes that the phosphorylation of a single S-16 AMELX site is critical for AMELX function.

Disruption of the phosphorylation of EMPs by native kinases also produces comparable phenotypes that are likely related to the lack of AMELX phosphorylation. Mutations in the Golgi kinase FAM20C which phosphorylates ECM proteins in mineralized tissues, including EMPs, and in its chaperon FAM20A^62^ cause syndromic AI characterized by thin, rodless enamel with a rough surface topography and ectopic calcifications.^63–65^ Similar phenotypes were observed in Fam20A and Fam20C KO mice,^48^ reinforcing the notion that EMP phosphorylation plays a critical role in controlling enamel mineralization and maintaining EO homeostasis. Likewise, mice lacking an AMELX fragment containing S16^47^ exhibit a similar enamel phenotype to that of our KI mouse model, further emphasizing the importance of AMELX phosphorylation for proper enamel formation.

Taken together, these findings indicate that the loss of AMELX phosphorylation produces a functional deficit similar to the complete loss of the entire AMELX protein. This highlights AMELX phosphorylation as a key regulatory event that couples mineralization with pH homeostasis and preserves ameloblast integrity during the secretory stage, when the ultrastructural framework of mature enamel is established. The mechanistic understanding of the importance of AMELX phosphorylation can be traced directly to the impact of this single post-translational modification on its effectiveness to transiently stabilize ACP and its subsequent transformation to CHA crystal to regulate the rate of enamel mineral deposition,^9^^,^^46^ which mechanistically serves to prevent the acidification of the enamel space to maintain ameloblast integrity and function. Accordingly, as we have demonstrated,^9^^,^^46^ phosphorylated AMELX effectively stabilizes the extracellular secretory stage environment, by restraining nascent enamel mineral formation and limiting proton release, thereby keeping the pH-regulatory demand within the physiological operating range of secretory ameloblasts. As shown in the present study, the absence of AMELX phosphorylation disrupts the extracellular regulation of enamel pH, leading to the dysregulation of the ameloblast pH regulation machinery. Future work will be needed to determine which components of the pH-regulatory network fail first under excess acid load (eg, bicarbonate generation vs transport vs secretion), and whether additional phosphorylation-dependent extracellular interactions among EMPs or ameloblast surface molecules contribute to cellular responses beyond mineralization control. In summary, our results expand our current understanding of the molecular mechanisms controlling amelogenesis, emphasizing the critical role of a single phosphorylation event in maintaining and coordinating EMP function, mineralization kinetics, and pH homeostasis to maintain ameloblast’s integrity and functional capacity.

## Supplementary Material

Bui_JBMR_Supplementary_material_f_ziag096

## Data Availability

All data are presented in the manuscript and in Supplementary material.
