# Supplementary material for: Enamel matrix acidification caused by loss of amelogenin phosphorylation disrupts ameloblast pH-regulatory machinery
Source: JBMR Plus. 2026 Jun 1;10(8):ziag096. doi: 10.1093/jbmrpl/ziag096 (PMC13332813; doi:10.1093/jbmrpl/ziag096)
Supplement: Bui_JBMR_Supplementary_material_f_ziag096 [file bui_jbmr_supplementary_material_f_ziag096.docx]

SUPPLEMENTARY MATERIAL

**Enamel matrix acidification caused by loss of AMELX phosphorylation disrupts ameloblast pH‑regulatory machinery.**

Ai Thu Bui^1,2,*^, Lasya Bhogadi^1,2^, Henry C Margolis^1,2,3^, Elia Beniash^1,2,§^.

1 – Department of Oral and Craniofacial Sciences, University of Pittsburgh School of Dental Medicine (UPSDM), Pittsburgh, PA, USA;

2 – Center for Craniofacial Regeneration, Pittsburgh, PA, USA;

3 – Department of Periodontics and Preventive Dentistry, UPSDM, Pittsburgh, PA, USA.

* First author, § Corresponding author: ebeniash@pitt.edu

**Materials and Methods**

Animals and Sample collection:

AmelxS16A KI (KI) mice on a C57BL/6 background were generated as previously described (1) and maintained at the University of Pittsburgh Division of Laboratory Animal Resources facility under approved protocols (#20108055, Institutional Animal Care and Use Committees, University of Pittsburgh). The animals were given a special diet consisting of hard chow (LabDiet, 5P76- Prolab Isopro RMH 3000) and soft gel (Diet Gel 76AGel, ClearH2O). Five-day-old to 9-day-old and twelve-week-old (12-wo) WT, HET and KI mice were euthanized with carbon dioxide, following decapitation, and their mandibles were collected for analyses, as described below and outlined in Suppl. Fig. S3.

Microdissection, RNA and protein extraction from mandibular incisors:

Incisors were isolated from mandibles of 12-wo mice by carefully removing bone from the mesial and distal sides of the jaw. Special care was taken to keep the labial surfaces of the incisors and the apical ends of the teeth intact. Mandibular incisal enamel organs (EO) were micro-dissected into five ~1-mm-wide segments corresponding to different stages of amelogenesis using molar positions as landmarks, as we have previously described (2) and shown in Fig. 1A. The segment closest to the apex, containing most of the secretory enamel, is labeled sec. The next segment is called the zone of active mineralization (zam). This segment is associated with the highest rate of enamel densification, which continues into mat1, and the attainment of full thickness of enamel (Fig. 1B). zam comprises a small portion of the late secretory stage, along with the transition and the earliest portion of the maturation stage (MS) (26). The next two segments contain mid- (mat1) and late- (mat2) maturation stages respectively, while the last segment (mat3) mainly consists of the protective cell layer. For each RT-qPCR reaction, EO segments of each kind were pooled from a minimum of 5 mice to obtain sufficient amounts of mRNA and the sample collection and the pooling procedure was repeated at least 3 times. A total of 40 male mice (WT and KI, 4 pooled samples of each segment), and 45 female mice (WT, HET and KI, 3 pooled samples from each segment) were used (Suppl Fig. S3).

To maximize yield and reduce the number of animals used, we have adopted and optimized a published protocol (47), which allowed us to extract sufficient quantities of protein and RNA from the same lysate obtained from five pooled strips of EO (see details in Suppl Fig. S3). The pooled EO strips were homogenized, using a Motorized Tissue Grinder and a RNAse-free pestle (Fisher Scientific), and lysed using ice-cold TRIzol® Reagent (Ambion, Life technologies). The lysates self-separate into three layers – containing RNA, DNA, and protein, respectively, from top to the bottom of the collection tube. The upper transparent phase containing RNA and the lower pinkish viscous phase containing protein were further purified and processed for mRNA and protein analyses, respectively (3) (Suppl Fig. S3).

Microdissection and protein extraction of the secretory enamel matrix from first molars of 5-day-old (-do) to 9-do mice:

To determine the relative quantities of extracellular Ca6 in secretory the enamel matrix (36), extracts from first molars (M1) of 5-do to 9-do mouse pups were used. The teeth were extracted under a dissecting microscope (Leica S8 APO, see details in Suppl. Fig. S3). EO, pulps and periodontium were removed by micro-dissection and cleaning with wet Kimwipe tissues (Kimberly-Clark, Roswell, GA). Enamel matrix protein isolation was then conducted according to a published procedure (48). Specifically, the enamel matrix and mineral were dissolved in cold 0.17 M HCl/0.98% formic acid and gently shaken for 2h at 4°C. Brief centrifugation (4000×g, 5min) was carried out to discard any residual dentin and undissolved materials. The supernatant was dialyzed using a 10 kDa cutoff dialysis membrane (Spectra/Por 6, MWCO 10kD, Spectrum Laboratories, Rancho Dominguez, USA) overnight against 500mL of 0.1% SDS. The dialyzed samples were separated into a colorless supernatant and a globular mass. The supernatant was then concentrated down to ~ 40µL by microcentrifugation using concentrator tubes (Amicon® Ultra-0.5mL, MWCO-3kDa, Millipore-Sigma Aldrich; #UFC500396) following manufacturer’s instructions. The concentrated supernatant was re-mixed with the globular mass, and the sample was dissolved completely in ~ 40µL of the Urea/SDS solution described above. The sample concentration was determined using Pierce^TM^ BCA protein assay kit (Thermo Scientific, #23227) and equal 10µg total protein aliquots per lane were loaded on a gradient NuPage 4-12% Bis-tris gel (Invitrogen, #NP0323BOX). The WBs were imaged and analyzed as described above. The OD of Ca6 was normalized using the total protein loaded on SDS PAGE stained with SimplyBlue^TM^ SafeStain (Invitrogen, #465034) and graphed as a ratio of the expression of WT vs KI EMPs.

RNA purification and RT-qPCR analysis:

RNA was extracted the pooled strips using chloroform and isoamyl alcohol (Sigma C0549) solutions (24:1), mixed in ratio 1:1 with 70% ethanol, and purified using a PureLink RNA Mini Kit (Invitrogen). Corresponding dissected segments from at least five mice were pooled to generate a minimum concentration of 20 ng/µL RNA, determined using a NanoDrop One C spectrophotometer (Thermo Scientific). Two-hundred (200) ng mRNA samples were transcribed (SuperScript IV enzyme, Thermo Fisher) for 20 min at 50°C and subjected to Real-time quantitative PCR (RT-qPCR) amplification by PowerTrack ™ SYBR GreenMasterMix (Thermo Fisher)and StepOne Plus Real Time PCR system (Applied Biosystems). mRNA expression levels of Ca2, Ca6, Ca9, Slc4a4, Slc24a4, Slc24a3 and Cftr in the EOs were assessed after normalization with β-actin (list of primers is presented in Suppl. Table S1).

Beside mRNA collection from dissected-strips of the EOs, whole EO layers were also collected. Sufficient quantities of 200 ng mRNA of whole EOs for subsequent analyses were collected by pooling enamel organs from mandibular incisors of 2 mice. A total of 14 male mice (8 WT and 6 KI) and 22 female mice (8 WT, 6 HET and 8 KI) were used in these experiments. Gene expression levels of Ca2, Ca6, Ca9 and Ca13 were determined relative to the expression level of β-actin, as described above.

Protein purification and Western-Blot analysis:

DNA fractions in TRIzol were carefully removed by tweezers and pipetting. Absolute ethanol was added to the remaining phenolic phase (volume ratio 1:1) and any residual DNA was precipitated by centrifugation for 5 minutes at 2000×g at 4°C. The phenol-ethanol supernatant was loaded into dialysis membranes and dialyzed against 500mL of 0.1% sodium dodecyl sulfate (SDS, Sigma-Aldrich, #71736). We have determined that 90% of the protein was in fraction P1, the globular milky fraction after dialysis, as shown in Suppl Fig. S4A. Therefore, only fraction P1 was further separated. The P1 fraction was then directly dissolved in Urea/SDS solution (5M Urea, 0.25M Tris-HCl and 0.5% SDS, final pH 8.0) and used for SDS-PAGE and Western blot (WB) analysis (Suppl Fig. S4, with corresponding molecular weight markers for proteins of interest). Since traces of Trizol after the dialysis and the addition of urea to dissolve the globular fraction can interfere with the BCA Protein Assay and the spectroscopic analysis using NanoDrop One C spectrophotometer, we were not able to determine the protein concentration before WB analyses. Therefore, equal volumes (10 µL) of each pooled sample were first loaded and analyzed by SDS-PAGE and WB, and the sample volume was latter readjusted using two references, β-actin and Tubulin (antibodies listed in Suppl. Table S2) for more accurate comparative analyses between different EO segments. The WB membranes were visualized using BIO-RAD ChemiDoc® MP Imaging system. The analysis of optical densities (OD) of WB bands was conducted using a Fiji software package (ImageJ V.1.53).

Since Ca2 is cytosolic and Ca9 is a membrane-bound Ca, we used the cellular components of the EOs for their analysis (12). For extracellular Ca6, an addition study was caried out using EMP extracts from 1st molars of 5- to 9-day old (-do) mice, corresponding to different stages of amelogenesis. These extracts were prepared as described in the preceding section on molar EMP extract preparation. OD of the bands containing Ca2, Ca9 and Ca6 were first normalized with two house-keeping proteins (β-actin and Tubulin) (Suppl. Table S2), and the protein levels in all 5 segments of both genotypes, were calculated as fractions of OD of the WT sec samples. Two male and two female pooled samples of each genotype (each containing at least 10 EOE strips dissected from 5 mice) were analyzed for Ca2 and Ca9. For Ca6, only one male and one female pooled sample were used due to the limited sample availability. Since the mat1-3 strips contain lesser amount of RNA and protein compared to sec and zam strips, double amounts of tissue from mat1-3 (equal to 10 mice) were pooled to have sufficient material for WB analysis.

Immunohistochemistry (IHC) and image analysis:

Mandibles of 8- to 12-wo adult male mice were excised, cleaned from soft tissues under cold PBS, fixed for two days in 10% buffered formalin (Fisher Chemicals, SF100-4) and decalcified for 4 weeks in 4.13% sodium EDTA (Fisher Bioreagents, BP120) at 4°C. After dehydration using an ethanol gradient, samples were processed (Leica ASP300S) and embedded in paraffin (Leica HistoEmbedder) and 7 µm thick sagittal sections were obtained using a microtome (Leica E61160). Immunofluorescent procedures were conducted then using a protocol developed in our laboratory ((49), see Suppl Table S2 for the list of antibodies and additional references for IF IHC procedures). Antigen retrieval in 10 mM sodium citrate buffer pH 6.0 (20 minutes at 95°C) was used prior to incubation with rabbit polyclonal anti-Ca2 antibodies (dilution 1:150) and rabbit polyclonal anti-Ca6 antibodies (dilution 1:150). Antigen retrieval with 0.25% trypsin/EDTA (15 minutes in 37°C) was used prior to incubation with rabbit monoclonal anti-Ca9 antibodies (dilution 1:250) levels. Sections were incubated with goat anti-rabbit secondary antibody (dilution 1:500). Auto-fluorescence was blocked using Vector TrueVIEW Autofluorescence Quenching kit (Vector Laboratories, SP-8400), and nuclei were counterstained with DAPI (2.5µM, 10 min). The sections were mounted with VectaShield® Plus Antifade mounting medium (Vector Laboratories #H-1900) and studied under a fluorescent microscope (Nikon ECLIPSE TE2000-E) equipped with NIS-Elements software. Immunofluorescence analysis was performed using Fiji image analysis package (ImageJ V.1.53). Three micrographs were taken from secretory stage and three from the maturation stage per sample and the channels containing immunofluorescence signals (green or red) were extracted from the images (Suppl. Fig. 6). Mean gray values (MGV) for immunofluorescence channels for triplicate images were calculated and their averages were used as MGVs per individual specimen. For each primary antibody, MGVs of three biological replicates of each genotype were obtained and mean MGV values were calculated. Relative strengths of the Ca9 and Ca2 fluorescent signals were calculated as a ratio of the mean MGVs of secretory and maturation stage ameloblasts of both genotypes to the mean MGV of the WT secretory ameloblasts. We also compared the mean MGVs of secretory and maturation ameloblasts to the mean MGVs of the stratum intermedium and papillary layers, respectively. Specifically, for each micrograph, 3 ROIs were selected, containing 5 ameloblasts with the corresponding stratum intermedium/papillary layer (based on DAPI staining of nuclei), as shown in Suppl Fig. S5C.

Determination of Ca activity of enamel organs and matrices using nitrophenol assay:

Freshly dissected EOs and secretory enamel matrix of four 12-wo mice of both genotypes and sexes were pooled separately and lysed in 1% NP40 cell lysis buffer (Invitrogen, #FNN0021). Eight (8) biological replicates from WT (4 males and 4 females) and 6 biological replicates from KI (3 males and 3 females) were studied. Protein extracts were adjusted to 1µg/µL concentration, based on BCA assay measurements, and aliquots of 10 µg per sample were stored at -80°C. Total Ca activity (50) was quantified using a colorimetric assay (Abcam, #ab284550), following the manufacturer instructions. To exclude non-CA-specific esterase activity, control samples were incubated with a Ca-specific inhibitor (20 mM Acetazolamide) for 30 minutes at 37°C, prior to adding of the substrate and the initiation of the reaction. The absorbance was measured at 405 nm, using a microplate reader (BioTek SYNERGY H1) and Gen5 2.07 software in the kinetic mode for 1 hour at 25°C. Absorbance data corresponding to the control samples with inhibitor were subtracted from the absorbance data representing the total esterase activity and converted to activity expressed in mU/mL, using a nitrophenol standard series, following the manufacturer’s instructions.

Statistical Analysis:

Quantitative data analysis was conducted using MS Excel 365 and Prism GraphPad 10 software packages. Normal data distribution was assessed using Shapiro-Wilk test, and the Grubb’s outlier test was performed to remove outliers. Statistical analyses were carried out using ANOVA with post-hoc Tukey’s corrections or Brown-Forsythe and Welch ANOVA test with Dunnett’s corrections, depending on the equal variance and distribution of each data set. Graphs were generated using OriginLab 2017, Prism GraphPad and CorelDraw software.

1. Shin, N.-Y., Yamazaki, H., Beniash, E., Yang, X., Margolis, S. S., Pugach, M. K. *et al.* (2020) Amelogenin phosphorylation regulates tooth enamel formation by stabilizing a transient amorphous mineral precursor *Journal of Biological Chemistry* **295**, 1943-1959

2. Bui, A. T., Lukashova, L., Verdelis, K., Vasquez, B., Bhogadi, L., Gabe, C. M. *et al.* (2023) Identification of stages of amelogenesis in the continuously growing mandiblular incisor of C57BL/6J male mice throughout life using molar teeth as landmarks *Front Physiol* **14**, 1144712

3. Hummon, A. B., Lim, S. R., Difilippantonio, M. J., and Ried, T. (2007) Isolation and solubilization of proteins after TRIzol extraction of RNA and DNA from patient material following prolonged storage *Biotechniques* **42**, 467-470, 472

1. Supplement Figure S1

| 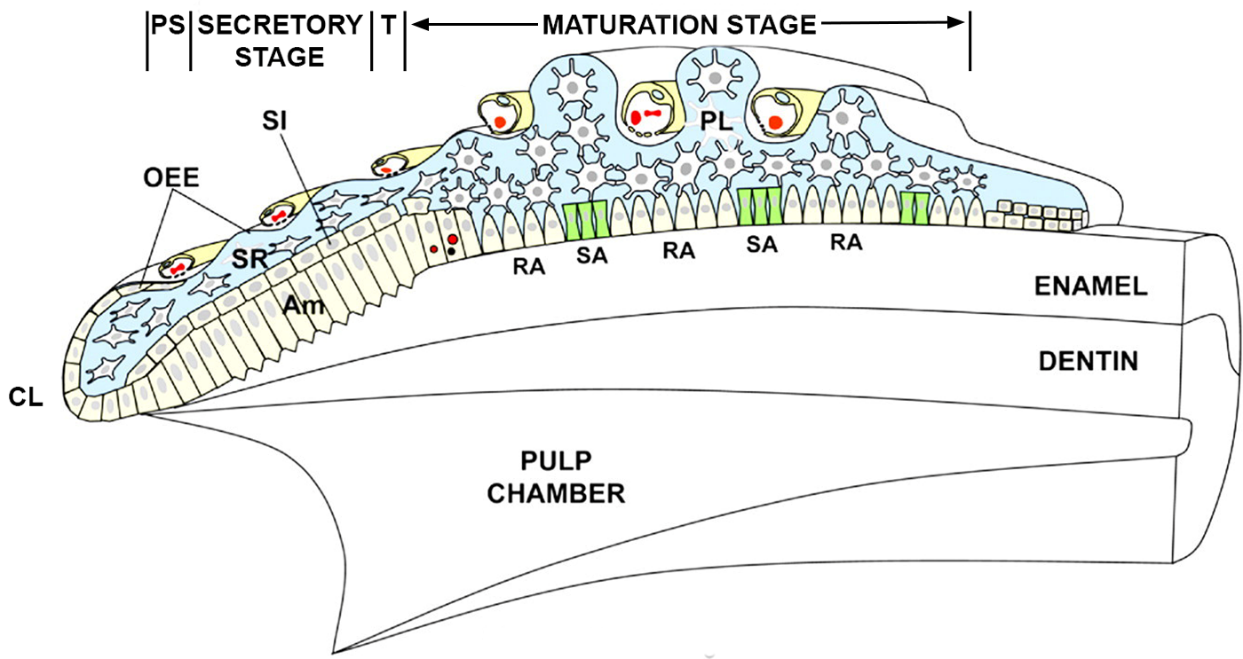 |
| --- |
| **Supplement Figure S1.** Schematic of the murine incisor enamel organ. Three stages of amelogenesis – presecretory (PS), secretory, transition (T) and maturation presented. Presecretory ameloblasts (PS) have characteristic columnar, habitus but lack Tomes’ processes. Secretory ameloblasts (Ab) develop Tomes’ processes. A thin layer of stratum intermedium (SI) abuts ameloblast layer. Stellate reticulum (SR) containing a network of star-like cells connected with long cell processes lays between SI and outer enamel epithelium (OEE) During the long-lasting process of enamel maturation, ameloblasts cyclically change structure and function between ruffle-ended ameloblasts (RA) and smooth-ended ameloblasts (SA). The SR and SI above the maturation stage ameloblast layer transform into a papillary layer (PL) which is highly vascularized. Cervical loop (CL) is the source of the enamel organ progenitor cells. Modified from Josephsen et al. (2010) Am J Physiol Cell Physiol 299, C1299-1307 with permission from APS. |

1. Supplement Figure S2

| 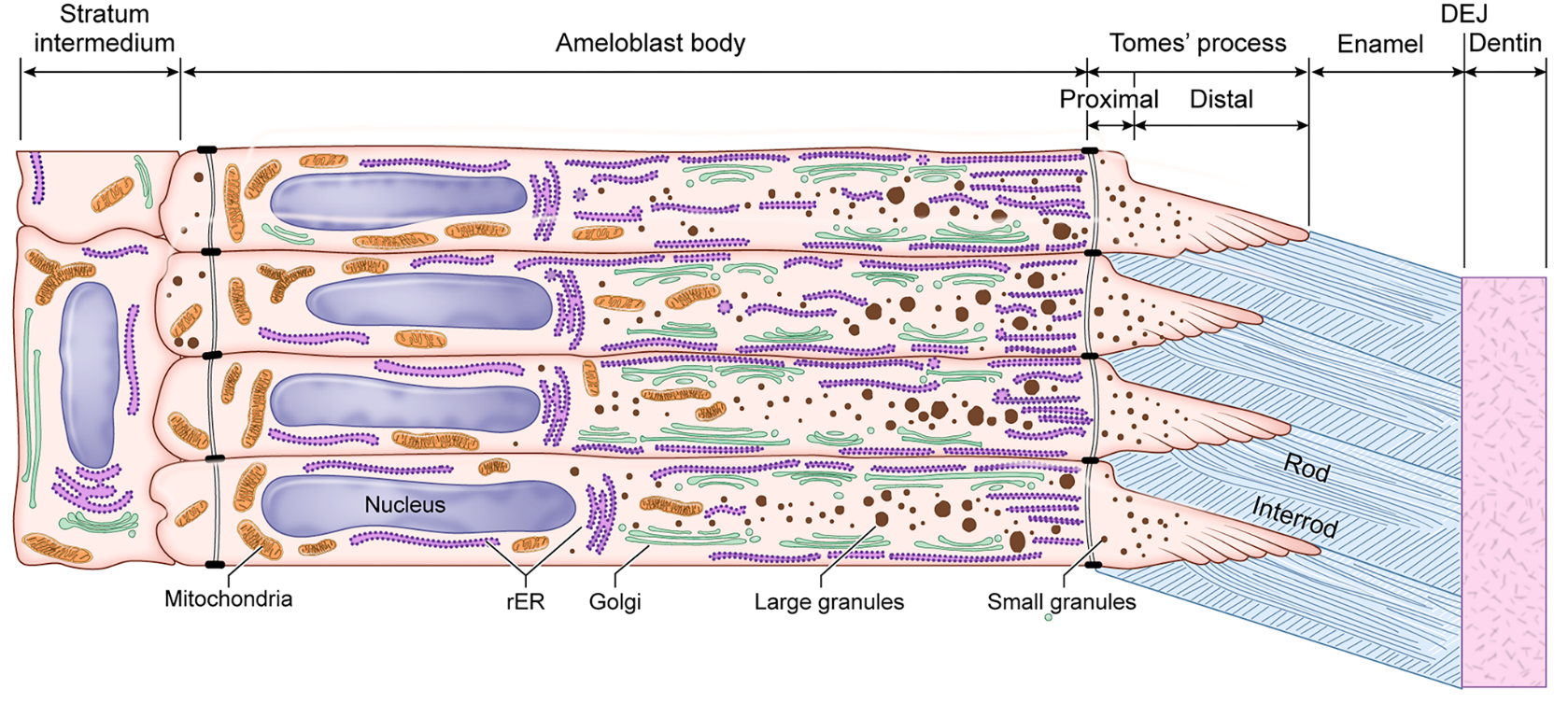 |
| --- |
| **Supplement Figure S2.** Schematic of the ultrastructural organization of secretory ameloblasts and their relationships with forming enamel rod and interrod. Secretory ameloblasts cells responsible for deposition of decussating enamel. Secretory ameloblasts are columnar polarized cells with the complex secretory apparatus on their distal end, called Tomes’ process. Tomes’ process is divided into the distal and proximal parts. Distal Tomes’ process produces one enamel rod, while the proximal Tomes’ process produces a portion of the interrod. Ameloblasts produce rod and interrod enamel while moving away from the dentino-enamel junction (DEJ). |

1. **Supplement Figure S3.** Experimental Procedures Workflow

| 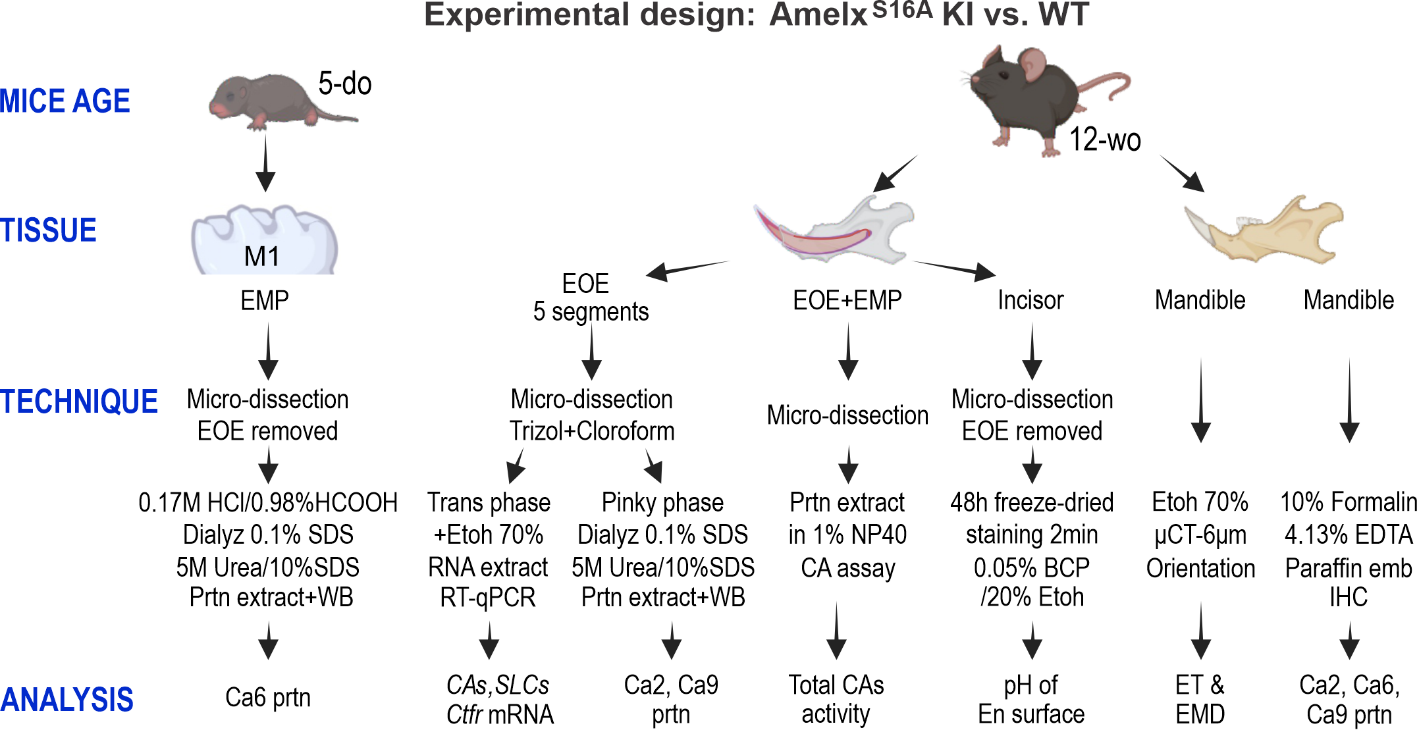 |
| --- |

1. Supplement Figure S4

| 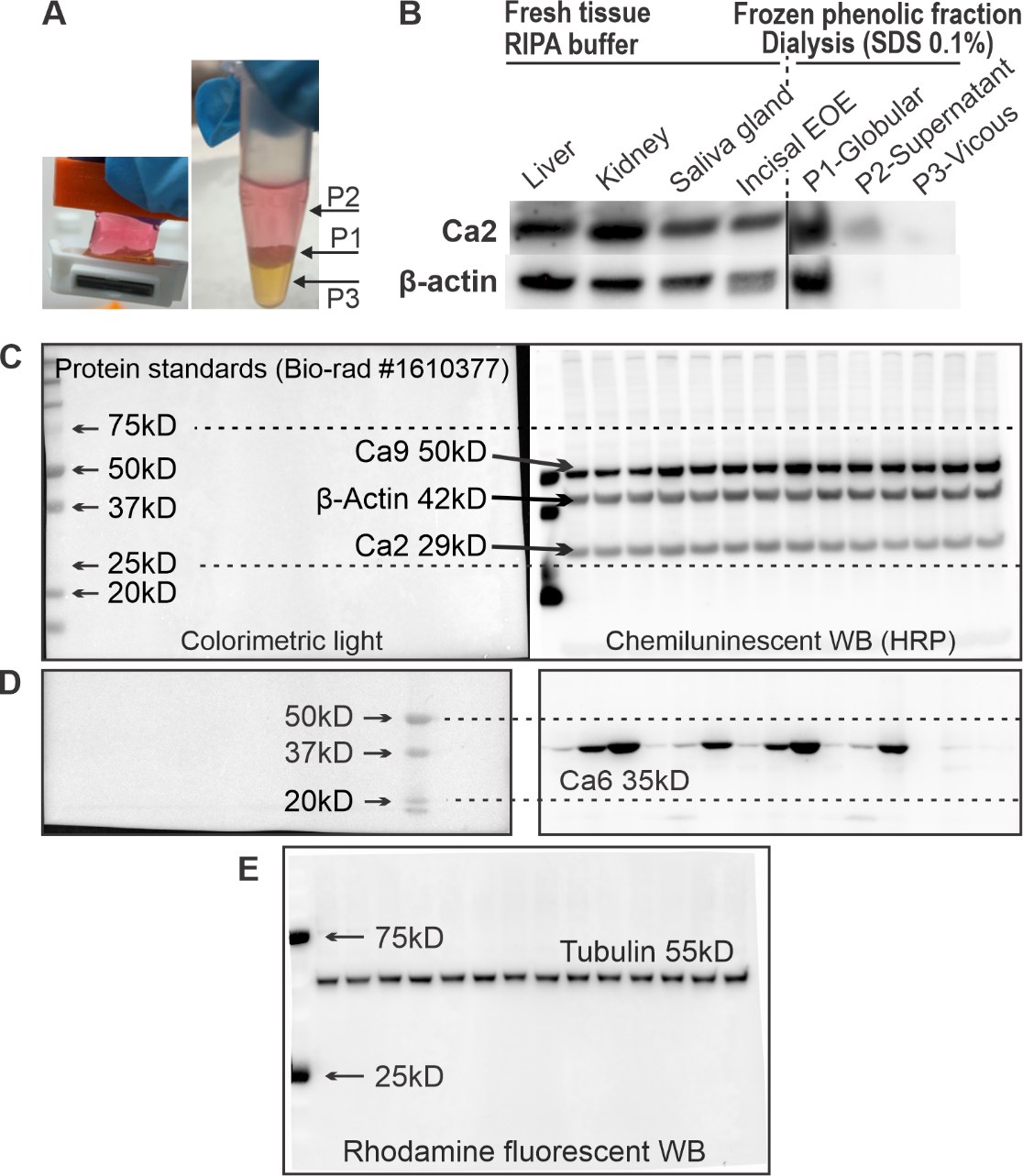 |
| --- |
| **Supplement Figure S4.** Protein purification from the phenol phase of Trizol following RNA extraction. (**A**) A phenol-ethanol supernatant was forming by adding absolute ethanol (1:1 volume ratio). Sample was loaded into Spectra/Por 6 regenerated cellulose dialysis membranes with 10kDa MWCO (Spectrum Laboratories, Rancho Dominguez, USA) and dialyzed against 500mL of 0.1% SDS (Sigma-Aldrich, #71736). This resulted in three distinct layers: P1, a milky globular middle layer, P2, a pinky transparent supernatant, and P3, a viscous bottom layer. (**B**) Western Blot analysis. Protein from each fraction were dissolved in Urea/SDS solution and concentrated as needed for SDS-PAGE. For comparison, 10µg of RIPA-extracted protein from liver, kidney, saliva gland and mandible incisal enamel organ were used as positive controls for carbonic anhydrase 2 (Ca2). Western blotting analysis revealed that P1 contained the majority of protein amount, meanwhile, P2 had only trace amounts and P3 showed no detectable signal for either Ca2 or the housekeeping protein β-actin. (**C-E**) Western Blot membrane showing bands of proteins of interest (Ca2, Ca6, Ca9) and house-keeping proteins (β-actin and Tubulin), along with their corresponding molecular weight markers. |

1. Supplement Figure S5

| 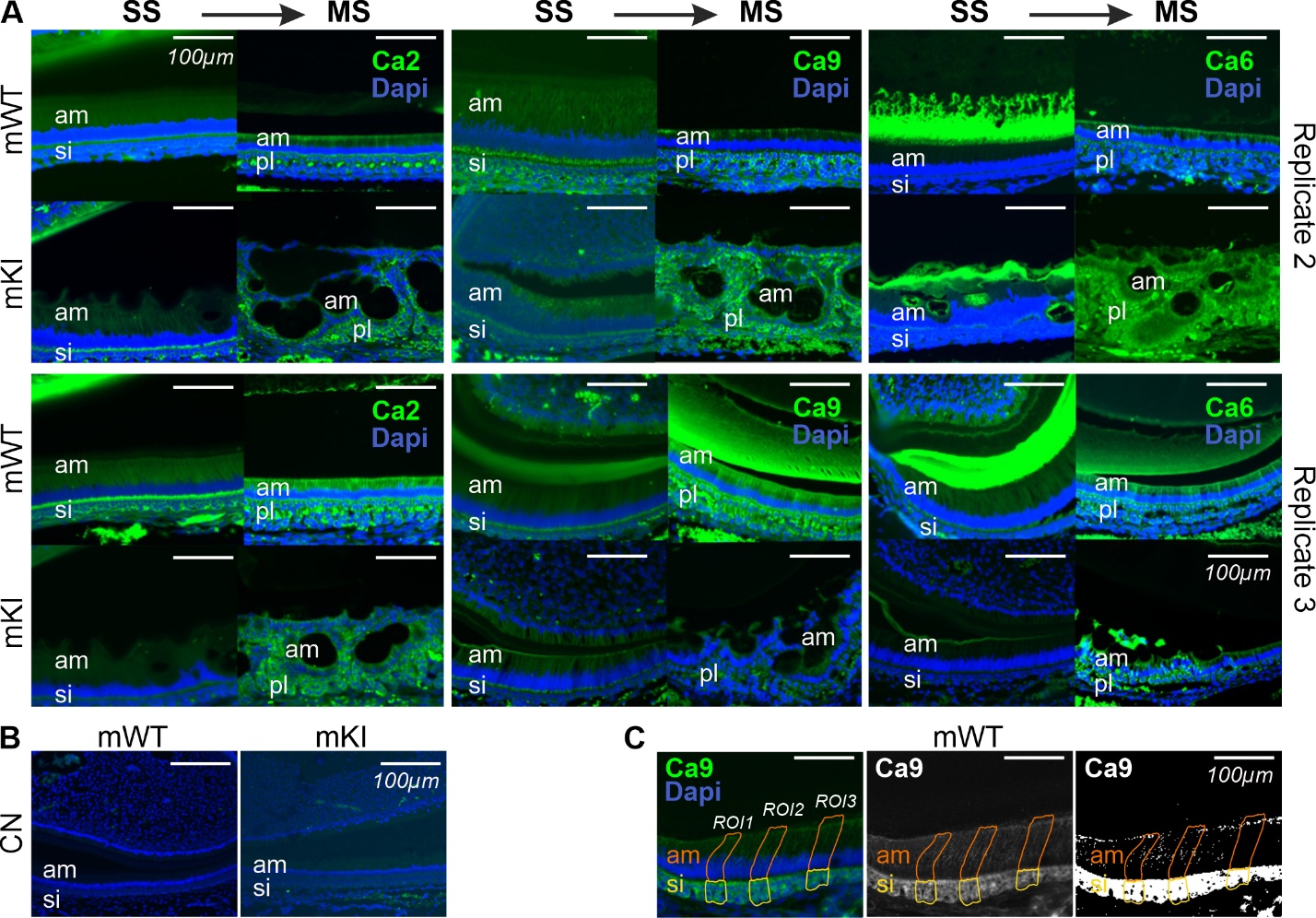 |
| --- |
| **Supplement Figure 5.** (**A**) Immunofluorescence (IF) staining of Ca2 (left panels), Ca9 (middle panels) and Ca6 (right panels) shows protein expression and localization detected in the enamel organ epithelium (EOE) of 8 to 12-week-old male mouse incisors. Two biological replicates were presented. Detection of green fluorescence signal indicating the protein of interest was focused on the ameloblast cell layer (am) and the stratum intermedium/papillary layer (si/pl), across two amelogenesis stages, the secretory (SS) and the maturation (MS). (**B**) Negative control using normal rabbit serum incubation was shown to confirm signal specificity in (A). (**C**) Representative image analysis illustrating the quantification method. Three regions of interest (ROIs) were randomly selected per section, each containing five ameloblasts and their corresponding si/pl layers. Fluorescence signals were converted to grayscale using ImageJ software, and mean grey values (MGVs) were calculated for am and si/pl in both SS and MS from WT and Amelx^S16A^ samples (scale bars: 100µm). |

1. Supplement Figure S6

| 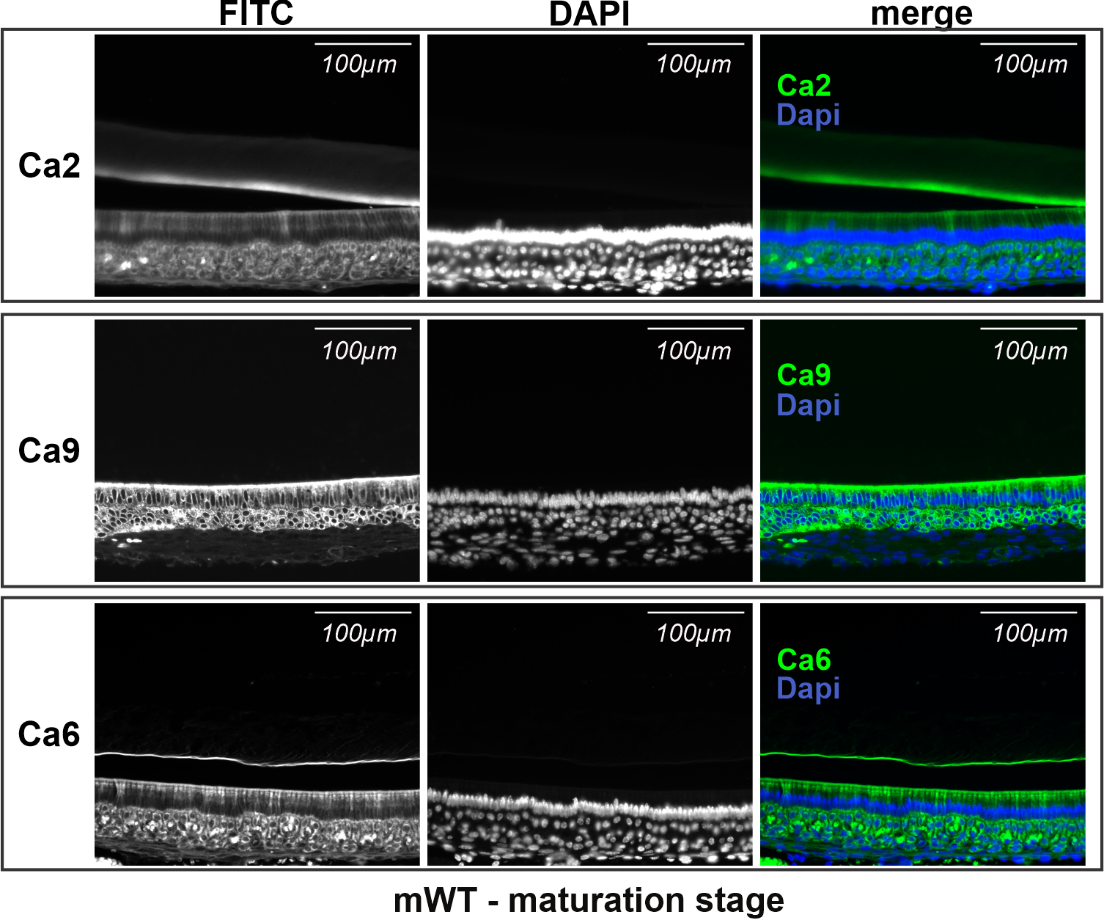 |
| --- |
| **Supplement Figure 6.** Immunofluorescence (IF) staining of Ca2 (top panels), Ca9 (middle panels) and Ca6 (bottom panels) showing protein expression level and distribution of the protein of interests in the maturation stage of a male WT incisor. Two different channels, FITC for the protein of interest, and DAPI, for the nucleus, are shown (scale bars: 100µm). |

1. **Supplement Table S1**. List of primers and its amplicon size used for RT-qPCR experiments.

| Gene | Amplicon size | Primer | Sequence |
| --- | --- | --- | --- |
| *Ca2*  (Carbonic Anhydrase 2) | 340 bp | F | CACAAGGCCTTCAGAAAGTCCTTGAAGC |
|  |  | R | GCTGCAGGGTTGTTTTACTTAAAGGACGC |
| *Ca6*  (Carbonic Anhydrase 6) | 220 bp | F | CTCTGTTATGGATCACAACAACAACACC |
|  |  | R | GCCAGTGTGTCATTTCCGGCTCC |
| *Ca9*  (Carbonic Anhydrase 9) | 305 bp | F | CCTTGTGGGGACCTCGTGATTC |
|  |  | R | GGTTTCTCCTTATTAGGCTCCAGTTTCTG |
| *SLC4a4*  (Solute carrier family 4, member 4) | 623 bp | F | CATGTGTGTGATGAAGAAGAAGTAGAAG |
|  |  | R | GACCGAAGGTTGGATTTCTTG |
| *SLC24a4*  (Solute carrier family 24, member 4) | 98 bp | F | GTCGCTCTCACTGTCCTTG |
|  |  | R | AAGCACAGGAAGACAGCATAG |
| *SLC24a3*  (Solute carrier family 24, member 3) | 105 bp | F | GTCTTTGTCACGGTCTTTGG |
|  |  | R | GATGGAGAAACAGAGGAACAC |
| *Cftr* (Cystic fibrosis transmembrane conductance regulator) | 291 bp | F | TGTTACACTCCATTCTTCACGCCCCTATGTC |
|  |  | R | TCCTGCCTTCAGATTCCAGTTGTTTGAGC |
| *β-actin*  (Beta actin) | 76 bp | F | GGGAAATCGTGCGTGACATC |
|  |  | R | GCGGCAGTGGCCATCTC |

1. **Supplement Table S2**. List of antibodies and additional references for IF IHC and Western Blot procedures.

| For IHC | | | | | | |
| --- | --- | --- | --- | --- | --- | --- |
| 1^st^ ab | Ref | Dilution | Antigen-retrieval | Blockage | 2^nd^ ab | Dilution |
| Rabbit polyclonal ab to **CA2** | Abcam,  #ab191343 | 1:150 | 10mM sodium citrate buffer pH6.0, 30 min in 95°C | (Yang et al., 2019) | Goat anti-Rabbit (Alexa Fluor® 488) (Invitrogen #A-11008) | 1:500 |
| Rabbit polyclonal ab to **CA6** | Biorbyt, #orb213629 | 1:150 | 10mM sodium citrate buffer pH6.0, 30 min in 95°C |  |  |  |
| Rabbit monoclonal ab to **CA9** | Abcam,  #ab243660 | 1:250 | 0.25%trypsin/EDTA, 15 min in 37°C |  |  |  |
| For Western Blot | | | | | | |
| 1^st^ ab | Ref | Dilution | Incubation | Blockage | Secondary | Dilution |
| Rabbit polyclonal ab to **CA2** | Abcam,  #ab191343 | 1:1500 | Overnight, 4°C | 5% BSA | Goat anti rabbit (HRP) (Abcam  #ab205718) | 1:5000 |
| Rabbit polyclonal ab to **CA6** | Biorbyt, #orb213629 | 1:1500 |  |  |  |  |
| Rabbit monoclonal ab to **CA9** | Abcam,  #ab243660 | 1:3000 |  |  |  |  |
| Mouse monoclonal ab to **β-Actin** | Abcam  #ab8226 | 1:3000 |  |  | Goat anti mouse (HRP) (Abcam  #ab205719) | 1:7000 |
| hFAB™ Rhodamine Anti-**Tubulin** | Bio-Rad  #12004165 | 1:700 | Room Temperature, 1.5 hour |  | N/A | N/A |

1. **Supplement Table S3.** RT-qPCR analysis of Ca2 mRNA expression level relative to β-actin mRNA level across five dissected segments of the enamel organ epithelium. Samples were collected from 12-week-old male and female mice of three genotypes: wild-type (WT), AmelxS16A Knock-in (KI) and heterozygous (HET). Statistical analysis was performed using three-way ANOVA (factors of segment, sex and genotype), followed by two-way ANOVA analysis and multiple post-hoc comparisons.

| ***Ca2* mRNA expression level (ratio to β-actin mRNA level) in EOE tissue** | | | | | | | | | | |  |
| --- | --- | --- | --- | --- | --- | --- | --- | --- | --- | --- | --- |
| Sex | Male | | | | Female | | | | | | |
| Genotype | WT (n=4) | | KI (n=4) | | WT (n=3) | | HET (n=3) | | KI (n=3) | | |
| Segment | Mean | SD | Mean | SD | Mean | SD | Mean | SD | Mean | SD |  |
| Sec | 0.15903 | 0.00946 | 0.05845 | 0.01112 | 0.16571 | 0.01779 | 0.06460 | 0.01480 | 0.04665 | 0.01796 |  |
| Zam | 0.24188 | 0.05019 | 0.29292 | 0.07037 | 0.33244 | 0.04281 | 0.31075 | 0.03681 | 0.23444 | 0.04835 |  |
| Mat1 | 0.38450 | 0.03328 | 0.35426 | 0.05043 | 0.44531 | 0.03457 | 0.27025 | 0.07453 | 0.25128 | 0.01320 |  |
| Mat2 | 0.31761 | 0.07055 | 0.41232 | 0.06782 | 0.45636 | 0.05406 | 0.32101 | 0.03823 | 0.25849 | 0.02318 |  |
| Mat3 | 0.23701 | 0.03492 | 0.24019 | 0.07379 | 0.25821 | 0.04071 | 0.17663 | 0.03236 | 0.14298 | 0.02647 |  |
| **Variances** | **p-value** | **Three-way ANOVA – between segment, sex and genotype** | | | | | | | | |  |
| Segment | <0.0001 | The population means of segments (sec, zam, mat1, mat2, and mat3) are significantly different | | | | | | | | |  |
| Sex | 0.3455 | The population means of sexes (male and female) are not significantly different | | | | | | | | |  |
| Genotype | <0.0001 | The population means of genotypes (WT and KI) are significantly different | | | | | | | | |  |
| Segment * Sex * Genotype | 0.0079 | The interaction between segment, sex and genotype is significantly different | | | | | | | | |  |
| **Combined both sexes** | | | | | | | | | | |  |
| Genotype | WT (n=7) | | HET (n=3) | | KI (n=7) | |  | | | |  |
| Segment | Mean | SD | Mean | SD | Mean | SD |  |  |  |  |  |
| Sec | 0.16190 | 0.01277 | 0.06460 | 0.01480 | 0.05339 | 0.01446 |  |  |  |  |  |
| Zam | 0.28069 | 0.06491 | 0.31075 | 0.03681 | 0.26786 | 0.06506 |  |  |  |  |  |
| Mat1 | 0.41056 | 0.04482 | 0.27025 | 0.07453 | 0.31013 | 0.06603 |  |  |  |  |  |
| Mat2 | 0.37707 | 0.09468 | 0.32101 | 0.03823 | 0.34640 | 0.09612 |  |  |  |  |  |
| Mat3 | 0.24609 | 0.03593 | 0.17663 | 0.03236 | 0.19853 | 0.07520 |  |  |  |  |  |
|  |  | **Tukey’s multiple comparisons test with two-way ANOVA – between segment and genotype** | | | | | | | | |  |
| *Comparison among genotypes of the same segment* | | | | | | | | | | |  |
| Groups | p-value | | Groups | | p-value | | Groups | | p-value | |  |
| Sec – WT vs. KI | 0.0040 | ** | Sec – WT vs. HET | | 0.0613 | ns | Sec – HET vs. KI | | 0.9618 | ns |  |
| Zam – WT vs. KI | 0.9185 | ns | Zam – WT vs. HET | | 0.7567 | ns | Zam – HET vs. KI | | 0.5685 | ns |  |
| Mat1 – WT vs. KI | 0.0083 | ** | Mat1 – WT vs. HET | | 0.0039 | ** | Mat1 – HET vs. KI | | 0.6134 | ns |  |
| Mat2 – WT vs. KI | 0.6174 | ns | Mat2 – WT vs. HET | | 0.3836 | ns | Mat2 – HET vs. KI | | 0.8194 | ns |  |
| Mat3 – WT vs. KI | 0.3181 | ns | Mat3 – WT vs. HET | | 0.2327 | ns | Mat3 – HET vs. KI | | 0.8621 | ns |  |
| *Comparison among segments of the same genotype* | | | | | | | | | | |  |
| Groups | p-value | | Groups | | p-value | | Groups | | p-value | |  |
| WT – sec vs. zam | 0.0046 | ** | HET – sec vs. zam | | <0.0001 | *** | KI – sec vs. zam | | <0.0001 | *** |  |
| WT – zam vs. mat1 | 0.0015 | *** | HET – zam vs. mat1 | | 0.9262 | ns | KI – zam vs. mat1 | | 0.6956 | ns |  |
| WT – mat1 vs. mat2 | 0.8428 | ns | HET – mat1 vs. mat2 | | 0.8465 | ns | KI – mat1 vs. mat2 | | 0.8004 | ns |  |
| WT – mat2 vs. mat3 | 0.0014 | ** | HET – mat2 vs. mat3 | | 0.0393 | * | KI – mat2 vs. mat3 | | 0.0002 | *** |  |
| WT – sec vs. mat1 | <0.0001 | *** | HET – sec vs. mat1 | | 0.0009 | *** | KI – sec vs. mat1 | | <0.0001 | *** |  |
| WT – zam vs. mat3 | 0.8264 | ns | HET – zam vs. mat3 | | 0.0659 | ns | KI – zam vs. mat3 | | 0.2221 | ns |  |

1. **Supplement Table S4.** Western blot analysis of Ca2 protein levels across five dissected segments of the enamel organ epithelium. Protein levels were normalized to two housekeeping proteins (β-actin and tubulin), and further to the expression level of WT sec segment. Samples were collected from 12-week-old male and female mice of two genotypes: wild-type (WT) and AmelxS16A Knock-in (KI). Statistical analysis was performed using two-way ANOVA (factors of segment and genotype), followed by multiple post-hoc comparisons.

| **Ca2 protein expression level (normalized with β-actin & Tubulin level)** | | | | **Two-way ANOVA – between segment and genotype** | | | | |
| --- | --- | --- | --- | --- | --- | --- | --- | --- |
| Genotype  Segment | WT | | KI | **Variances** | **p-value** | **Significance** | | |
|  | Mean ± SD (n) | | Mean ± SD (n) | Segment | 0.3424 | The population means of segments (sec, zam, mat) are not significantly different | | |
| Sec | 1.0000 ± 0.0352 (8) | | 0.7926 ± 0.1476 (8) |  |  |  |  |  |
| Zam | 0.8724 ± 0.0905 (4) | | 1.0133 ± 0.2285 (4) | Genotype | <0.0001 | The population means of genotypes (WT and KI) are significantly different | | |
| Mat1 | 0.6964 ± 0.1081 (4) | | 1.2447 ± 0.3189 (4) |  |  |  |  |  |
| Mat2 | 0.7491 ± 0.1229 (4) | | 1.2962 ± 0.3989 (4) | Segment * Genotype | <0.0001 | The interaction between segment and genotype is significantly different | | |
| Mat3 | 0.8364 ± 0.1793 (4) | | 1.2717 ± 0.1678 (4) |  |  |  |  |  |
| **Tukey’s multiple comparisons test with two-way ANOVA – between segment and genotype** | | | | | | | | |
| Groups | p-value | | Groups | p-value | | Groups | p-value | |
| Sec – WT vs. KI | 0.0361 | * | WT – sec vs. zam | 0.8100 | ns | KI – sec vs. zam | 0.3416 | ns |
| Zam – WT vs. KI | 0.3036 | ns | WT – zam vs. mat1 | 0.6907 | ns | KI – zam vs. mat1 | 0.4378 | ns |
| Mat1 – WT vs. KI | 0.0002 | *** | WT – mat1 vs. mat2 | 0.9949 | ns | KI – mat1 vs. mat2 | 0.9953 | ns |
| Mat2 – WT vs. KI | 0.0002 | *** | WT – mat2 vs. mat3 | 0.9663 | ns | KI – mat2 vs. mat3 | 0.9997 | ns |
| Mat3 – WT vs. KI | 0.0026 | ** | WT – sec vs. mat1 | 0.0913 | ns | KI – sec vs. mat1 | 0.0036 | ** |
|  |  |  | WT – zam vs. mat3 | 0.9988 | ns | KI – zam vs. mat3 | 0.3276 | ns |

1. **Supplement Table S5.** RT-qPCR analysis of Ca2 mRNA expression level across the whole enamel organ epithelium layer. The expression level of Ca2 were normalized to β-actin mRNA level. Samples were collected from 12-week-old male and female mice of three genotypes: wild-type (WT), AmelxS16A Knock-in (KI) and heterozygous (HET). Statistical analysis was performed using two-way ANOVA (factors of sex and genotype), followed by multiple post-hoc comparisons.

| ***Ca2* mRNA expression level (ratio to β-actin mRNA) in EOE tissue** | | | | | | | **Two-way ANOVA – between sex and genotype** | | | | |
| --- | --- | --- | --- | --- | --- | --- | --- | --- | --- | --- | --- |
| Sex | | Male | | Female | | | **Variances** | **p-value** | **Significance** | | |
| Genotype | | WT | KI | WT | HET | KI | Sex | 0.4465 | The population means of sexes (m, f) are not significantly different | | |
| Sample 1 | | 0.50589 | 0.45426 | 0.58725 | 0.48506 | 0.36836 |  |  |  |  |  |
| Sample 2 | | 0.43469 | 0.44422 | 0.66194 | 0.38890 | 0.36740 | Genotype | 0.0001 | The population means of genotypes are significantly different | | |
| Sample 3 | | 0.42224 | 0.45324 | 0.53440 | 0.39833 | 0.33349 |  |  |  |  |  |
| Sample 4 | | 0.50252 |  |  |  | 0.35240 | Sex * Genotype | 0.0003 | The interaction between sex and genotype is significantly different | | |
| Mean | | 0.46634 | 0.45057 | 0.59453 | 0.42410 | 0.35541 |  |  |  |  |  |
| SD | | 0.04405 | 0.00552 | 0.06408 | 0.05301 | 0.01634 |  |  |  |  |  |
| **Combined both sexes** | | | | | | | **Tukey’s multiple comparisons test with one-way ANOVA** | | | | |
| Genotype | WT (n=7) | | HET (n=3) | | KI (n=7) | | Groups | | | p-value | |
| Mean±SD | 0.52128 ± 0.08387 | | 0.42410 ± 0.05301 | | 0.39620 ± 0.05226 | | WT vs. KI | | | 0.0101 | * |
|  | | | | | | | WT vs. HET | | | 0.1302 | ns |
|  |  |  |  |  |  |  | HET vs. KI | | | 0.8239 | ns |

1. **Supplement Table S6.** RT-qPCR analysis of Ca9 mRNA expression level relative to β-actin mRNA level across five dissected segments of the enamel organ epithelium. Samples were collected from 12-week-old male and female mice of three genotypes: wild-type (WT), AmelxS16A Knock-in (KI) and heterozygous (HET). Statistical analysis was performed using three-way ANOVA (factors of segment, sex and genotype), followed by two-way ANOVA analysis and multiple post-hoc comparisons.

| ***Ca9* mRNA expression level (ratio to β-actin mRNA level) in EOE tissue** | | | | | | | | | | |  |
| --- | --- | --- | --- | --- | --- | --- | --- | --- | --- | --- | --- |
| Sex | Male | | | | Female | | | | | | |
| Genotype | WT (n=5) | | KI (n=4) | | WT (n=4) | | HET (n=3) | | KI (n=4) | | |
| Segment | Mean | SD | Mean | SD | Mean | SD | Mean | SD | Mean | SD |  |
| Sec | 0.00404 | 0.00191 | 0.00284 | 0.00153 | 0.00432 | 0.00085 | 0.00212 | 0.00018 | 0.00240 | 0.00078 |  |
| Zam | 0.00077 | 0.00045 | 0.00280 | 0.00127 | 0.00099 | 0.00013 | 0.00162 | 0.00041 | 0.00253 | 0.00046 |  |
| Mat1 | 0.00062 | 0.00038 | 0.00086 | 0.00052 | 0.00057 | 0.00015 | 0.00074 | 0.00032 | 0.00111 | 0.00022 |  |
| Mat2 | 0.00075 | 0.00040 | 0.00086 | 0.00036 | 0.00064 | 0.00016 | 0.00063 | 0.00009 | 0.00120 | 0.00039 |  |
| Mat3 | 0.00058 | 0.00031 | 0.00088 | 0.00034 | 0.00057 | 0.00016 | 0.00073 | 0.00017 | 0.00079 | 0.00030 |  |
| **Variances** | **p-value** | **Three-way ANOVA – between segment, sex and genotype** | | | | | | | | |  |
| Segment | <0.0001 | The population means of segments (sec, zam, mat1, mat2, and mat3) are significantly different | | | | | | | | |  |
| Sex | 0.9444 | The population means of sexes (male and female) are not significantly different | | | | | | | | |  |
| Genotype | 0.2047 | The population means of genotypes (WT and KI) are not significantly different | | | | | | | | |  |
| Segment * Genotype | <0.0001 | The interaction between segment and genotype is significantly different | | | | | | | | |  |
| Segment * Sex * Genotype | 0.8358 | The interaction between segment, sex and genotype is not significantly different | | | | | | | | |  |
| **Combined both sexes** | | | | | | | | | | |  |
| Genotype | WT (n=9) | | HET (n=3) | | KI (n=8) | |  | | | |  |
| Segment | Mean | SD | Mean | SD | Mean | SD |  |  |  |  |  |
| Sec | 0.00418 | 0.00138 | 0.00212 | 0.00018 | 0.00259 | 0.00107 |  |  |  |  |  |
| Zam | 0.00105 | 0.00069 | 0.00162 | 0.00041 | 0.00266 | 0.00090 |  |  |  |  |  |
| Mat1 | 0.00060 | 0.00029 | 0.00074 | 0.00032 | 0.00098 | 0.00039 |  |  |  |  |  |
| Mat2 | 0.00070 | 0.00031 | 0.00063 | 0.00009 | 0.00103 | 0.00039 |  |  |  |  |  |
| Mat3 | 0.00058 | 0.00024 | 0.00073 | 0.00017 | 0.00083 | 0.00030 |  |  |  |  |  |
|  |  | **Tukey’s multiple comparisons test with two-way ANOVA – between segment and genotype** | | | | | | | | |  |
| *Comparison among genotypes of the same segment* | | | | | | | | | | |  |
| Groups | p-value | | Groups | | p-value | | Groups | | p-value | |  |
| Sec – WT vs. KI | <0.0001 | *** | Sec – WT vs. HET | | <0.0001 | *** | Sec – HET vs. KI | | 0.5509 | ns |  |
| Zam – WT vs. KI | <0.0001 | *** | Zam – WT vs. HET | | 0.3923 | ns | Zam – HET vs. KI | | 0.0516 | ns |  |
| Mat1 – WT vs. KI | 0.4408 | ns | Mat1 – WT vs. HET | | 0.9455 | ns | Mat1 – HET vs. KI | | 0.8382 | ns |  |
| Mat2 – WT vs. KI | 0.5579 | ns | Mat2 – WT vs. HET | | 0.9849 | ns | Mat2 – HET vs. KI | | 0.6383 | ns |  |
| Mat3 – WT vs. KI | 0.7008 | ns | Mat3 – WT vs. HET | | 0.9384 | ns | Mat3 – HET vs. KI | | 0.9679 | ns |  |
| *Comparison among segments of the same genotype* | | | | | | | | | | |  |
| Groups | p-value | | Groups | | p-value | | Groups | | p-value | |  |
| WT – sec vs. zam | <0.0001 | *** | HET – sec vs. zam | | 0.8829 | ns | KI – sec vs. zam | | 0.9993 | ns |  |
| WT – zam vs. mat1 | 0.5717 | ns | HET – zam vs. mat1 | | 0.4569 | ns | KI – zam vs. mat1 | | <0.0001 | *** |  |
| WT – mat1 vs. mat2 | 0.9972 | ns | HET – mat1 vs. mat2 | | 0.9996 | ns | KI – mat1 vs. mat2 | | >0.9999 | ns |  |
| WT – mat2 vs. mat3 | 0.9947 | ns | HET – mat2 vs. mat3 | | 0.9997 | ns | KI – mat2 vs. mat3 | | 0.9755 | ns |  |
| WT – sec vs. mat1 | <0.0001 | *** | HET – sec vs. mat1 | | 0.0786 | ns | KI – sec vs. mat1 | | <0.0001 | *** |  |
| WT – zam vs. mat3 | 0.5340 | ns | HET – zam vs. mat3 | | 0.4465 | ns | KI – zam vs. mat3 | | <0.0001 | *** |  |

1. **Supplement Table S7.** Western blot analysis of Ca9 protein levels across five dissected segments of the enamel organ epithelium. Protein levels were normalized to two housekeeping proteins (β-actin and tubulin), and further to the expression level of WT sec segment. Samples were collected from 12-week-old male and female mice of two genotypes: wild-type (WT) and AmelxS16A Knock-in (KI). Statistical analysis was performed using two-way ANOVA (factors of segment and genotype), followed by multiple post-hoc comparisons.

| **Ca9 protein expression level (normalized with β-actin & Tubulin level)** | | | | **Two-way ANOVA – between segment and genotype** | | | | |
| --- | --- | --- | --- | --- | --- | --- | --- | --- |
| Genotype  Segment | WT | | KI | **Variances** | **p-value** | **Significance** | | |
|  | Mean ± SD (n) | | Mean ± SD (n) | Segment | <0.0001 | The population means of segments (sec, zam, mat) are significantly different | | |
| Sec | 1.0000 ± 0.0568 (8) | | 0.6175 ± 0.0926 (8) |  |  |  |  |  |
| Zam | 1.5777 ± 0.3156 (4) | | 1.0659 ± 0.1865 (4) | Genotype | <0.0001 | The population means of genotypes (WT and KI) are significantly different | | |
| Mat1 | 1.8768 ± 0.3882 (4) | | 1.2053 ± 0.0902 (4) |  |  |  |  |  |
| Mat2 | 1.7237 ± 0.3291 (4) | | 1.0170 ± 0.1899 (4) | Segment * Genotype | 0.1581 | The interaction between segment and genotype is not significantly different | | |
| Mat3 | 1.0244 ± 0.0985 (4) | | 0.7138 ± 0.1198 (4) |  |  |  |  |  |
| **Tukey’s multiple comparisons test with two-way ANOVA – between segment and genotype** | | | | | | | | |
| Groups | p-value | | Groups | p-value | | Groups | p-value | |
| Sec – WT vs. KI | 0.0004 | *** | WT – sec vs. zam | 0.0002 | *** | KI – sec vs. zam | 0.0054 | ** |
| Zam – WT vs. KI | 0.0007 | *** | WT – zam vs. mat1 | 0.2198 | ns | KI – zam vs. mat1 | 0.8522 | ns |
| Mat1 – WT vs. KI | <0.0001 | *** | WT – mat1 vs. mat2 | 0.8045 | ns | KI – mat1 vs. mat2 | 0.6591 | ns |
| Mat2 – WT vs. KI | <0.0001 | *** | WT – mat2 vs. mat3 | 0.0001 | *** | KI – mat2 vs. mat3 | 0.2085 | ns |
| Mat3 – WT vs. KI | 0.0314 | * | WT – sec vs. mat1 | <0.0001 | *** | KI – sec vs. mat1 | 0.0002 | *** |
|  |  |  | WT – zam vs. mat3 | 0.0026 | ** | KI – zam vs. mat3 | 0.1043 | ns |

1. **Supplement Table S8.** RT-qPCR analysis of Ca9 mRNA expression level across the whole enamel organ epithelium layer. The expression level of Ca9 were normalized to β-actin mRNA level. Samples were collected from 12-week-old male and female mice of three genotypes: wild-type (WT), AmelxS16A Knock-in (KI) and heterozygous (HET). Statistical analysis was performed using two-way ANOVA (factors of sex and genotype), followed by multiple post-hoc comparisons.

| ***Ca9* mRNA expression level (ratio to β-actin mRNA) in EOE tissue** | | | | | | | **Two-way ANOVA – between sex and genotype** | | | | |
| --- | --- | --- | --- | --- | --- | --- | --- | --- | --- | --- | --- |
| Sex | | Male | | Female | | | **Variances** | **p-value** | **Significance** | | |
| Genotype | | WT | KI | WT | HET | KI | Sex | 0.0321 | The population means of sexes (m, f) are significantly different | | |
| Sample 1 | | 0.00124 | 0.00163 | 0.00177 | 0.00140 | 0.00143 |  |  |  |  |  |
| Sample 2 | | 0.00129 | 0.00144 | 0.00131 | 0.00206 | 0.00133 | Genotype | 0.1336 | The population means of genotypes are not significantly different | | |
| Sample 3 | | 0.00108 | 0.00149 | 0.00160 | 0.00138 | 0.00157 |  |  |  |  |  |
| Sample 4 | | 0.00110 |  | 0.00169 |  | 0.00182 | Sex * Genotype | 0.0434 | The interaction between sex and genotype is significantly different | | |
| Mean | | 0.00118 | 0.00152 | 0.00159 | 0.00161 | 0.00154 |  |  |  |  |  |
| SD | | 0.00010 | 0.00010 | 0.00020 | 0.00039 | 0.00021 |  |  |  |  |  |
| **Combined both sexes** | | | | | | | **Tukey’s multiple comparisons test with one-way ANOVA** | | | | |
| Genotype | WT (n=8) | | HET (n=3) | | KI (n=7) | | Groups | | | p-value | |
| Mean±SD | 0.00139 ± 0.00027 | | 0.00161 ± 0.00039 | | 0.00153 ± 0.00016 | | WT vs. KI | | | 0.5316 | ns |
|  | | | | | | | WT vs. HET | | | 0.4003 | ns |
|  |  |  |  |  |  |  | HET vs. KI | | | 0.8793 | ns |

1. **Supplement Table S9.** RT-qPCR analysis of Ca6 mRNA expression level relative to β-actin mRNA level across five dissected segments of the enamel organ epithelium. Samples were collected from 12-week-old male and female mice of three genotypes: wild-type (WT), AmelxS16A Knock-in (KI) and heterozygous (HET). Statistical analysis was performed using three-way ANOVA (factors of segment, sex and genotype), followed by two-way ANOVA analysis and multiple post-hoc comparisons.

| ***Ca6* mRNA expression level (ratio to β-actin mRNA level) in EOE tissue** | | | | | | | | | | |  |
| --- | --- | --- | --- | --- | --- | --- | --- | --- | --- | --- | --- |
| Sex | Male | | | | Female | | | | | | |
| Genotype | WT (n=4) | | KI (n=4) | | WT (n=3) | | HET (n=3) | | KI (n=3) | | |
| Segment | Mean | SD | Mean | SD | Mean | SD | Mean | SD | Mean | SD |  |
| Sec | 0.33279 | 0.08029 | 0.00373 | 0.00187 | 0.26819 | 0.07192 | 0.05370 | 0.03051 | 0.00777 | 0.00888 |  |
| Zam | 0.48935 | 0.09867 | 0.31776 | 0.01556 | 0.64649 | 0.12650 | 0.58244 | 0.08191 | 0.36682 | 0.01685 |  |
| Mat1 | 0.02194 | 0.01106 | 0.04229 | 0.01454 | 0.01834 | 0.00523 | 0.05985 | 0.01841 | 0.02270 | 0.02646 |  |
| Mat2 | 0.00192 | 0.00119 | 0.00227 | 0.00159 | 0.00205 | 0.00037 | 0.00454 | 0.00208 | 0.01212 | 0.02065 |  |
| Mat3 | 0.00225 | 0.00157 | 0.00055 | 0.00035 | 0.00064 | 0.00036 | 0.00290 | 0.00242 | 0.00083 | 0.00045 |  |
| **Variances** | **p-value** | **Three-way ANOVA – between segment, sex and genotype** | | | | | | | | |  |
| Segment | <0.0001 | The population means of segments (sec, zam, mat1, mat2, and mat3) are significantly different | | | | | | | | |  |
| Sex | 0.2205 | The population means of sexes (male and female) are not significantly different | | | | | | | | |  |
| Genotype | <0.0001 | The population means of genotypes (WT and KI) are significantly different | | | | | | | | |  |
| Segment * Genotype | <0.0001 | The interaction between segment and genotype is significantly different | | | | | | | | |  |
| Segment * Sex * Genotype | 0.1374 | The interaction between segment, sex and genotype is not significantly different | | | | | | | | |  |
| **Combined both sexes** | | | | | | | | | | |  |
| Genotype | WT (n=7) | | HET (n=3) | | KI (n=7) | |  | | | |  |
| Segment | Mean | SD | Mean | SD | Mean | SD |  |  |  |  |  |
| Sec | 0.30510 | 0.07836 | 0.05370 | 0.03051 | 0.00575 | 0.00632 |  |  |  |  |  |
| Zam | 0.55669 | 0.13137 | 0.58244 | 0.08191 | 0.34229 | 0.03054 |  |  |  |  |  |
| Mat1 | 0.02014 | 0.00798 | 0.05985 | 0.01841 | 0.03389 | 0.02119 |  |  |  |  |  |
| Mat2 | 0.00197 | 0.00087 | 0.00454 | 0.00208 | 0.00719 | 0.01454 |  |  |  |  |  |
| Mat3 | 0.00156 | 0.00142 | 0.00290 | 0.00242 | 0.00069 | 0.00040 |  |  |  |  |  |
|  |  | **Tukey’s multiple comparisons test with two-way ANOVA – between segment and genotype** | | | | | | | | |  |
| *Comparison among genotypes of the same segment* | | | | | | | | | | |  |
| Groups | p-value | | Groups | | p-value | | Groups | | p-value | |  |
| Sec – WT vs. KI | <0.0001 | *** | Sec – WT vs. HET | | <0.0001 | *** | Sec – HET vs. KI | | 0.3136 | ns |  |
| Zam – WT vs. KI | <0.0001 | *** | Zam – WT vs. HET | | 0.7214 | ns | Zam – HET vs. KI | | <0.0001 | *** |  |
| Mat1 – WT vs. KI | 0.8660 | ns | Mat1 – WT vs. HET | | 0.4796 | ns | Mat1 – HET vs. KI | | 0.7174 | ns |  |
| Mat2 – WT vs. KI | 0.9763 | ns | Mat2 – WT vs. HET | | 0.9967 | ns | Mat2 – HET vs. KI | | 0.9964 | ns |  |
| Mat3 – WT vs. KI | 0.9993 | ns | Mat3 – WT vs. HET | | 0.9991 | ns | Mat3 – HET vs. KI | | 0.9975 | ns |  |
| *Comparison among segments of the same genotype* | | | | | | | | | | |  |
| Groups | p-value | | Groups | | p-value | | Groups | | p-value | |  |
| WT – sec vs. zam | <0.0001 | *** | HET – sec vs. zam | | <0.0001 | *** | KI – sec vs. zam | | <0.0001 | *** |  |
| WT – zam vs. mat1 | <0.0001 | *** | HET – zam vs. mat1 | | <0.0001 | *** | KI – zam vs. mat1 | | <0.0001 | *** |  |
| WT – mat1 vs. mat2 | 0.9611 | ns | HET – mat1 vs. mat2 | | 0.6286 | ns | KI – mat1 vs. mat2 | | 0.8224 | ns |  |
| WT – mat2 vs. mat3 | >0.9999 | ns | HET – mat2 vs. mat3 | | >0.9999 | ns | KI – mat2 vs. mat3 | | 0.9998 | ns |  |
| WT – sec vs. mat1 | <0.0001 | *** | HET – sec vs. mat1 | | 0.9999 | ns | KI – sec vs. mat1 | | 0.7928 | ns |  |
| WT – zam vs. mat3 | <0.0001 | *** | HET – zam vs. mat3 | | <0.0001 | *** | KI – zam vs. mat3 | | <0.0001 | *** |  |

1. **Supplement Table S10.** Western blot analysis of Ca6 protein levels in the molar enamel matrix from 5- to 9-day-old (-do) mice, representing secretory, transition and maturation stages of amelogenesis. Protein levels were normalized to total protein loaded on SDS PAGE stained with SimplyBlueTM SafeStain (Invitrogen, #465034), and further to the expression level in the enamel matrix of 5-do WT mice. Samples were collected from two genotypes: wild-type (WT) and AmelxS16A Knock-in (KI). Statistical analysis was performed using two-way ANOVA (factors of age and genotype), followed by multiple post-hoc comparisons.

| **Ca6 protein expression level (normalized to total loading protein level)** | | | | **Two-way ANOVA – between age and genotype** | | | | |
| --- | --- | --- | --- | --- | --- | --- | --- | --- |
| Genotype  Age | WT | | KI | **Variances** | **p-value** | **Significance** | | |
|  | Mean ± SD (n) | | Mean ± SD (n) | Age | 0.0631 | The population means of mouse ages (5 to 9-do) are not significantly different | | |
| 5-do (≈ Secretory) | 1.0000 ± 0.1618 (6) | | 0.2956 ± 0.1148 (7) |  |  |  |  |  |
| 6-do (≈ Late secretory) | 1.0314 ± 0.0272 (3) | | 0.2739 ± 0.0179 (3) | Genotype | <0.0001 | The population means of genotypes (WT and KI) are significantly different | | |
| 7-do (≈ Transition) | 1.0249 ± 0.4445 (3) | | 0.2859 ± 0.0670 (3) |  |  |  |  |  |
| 8-do (≈ Early Maturation) | 0.7670 ± 0.1915 (3) | | 0.2323 ± 0.1101 (3) | Age * Genotype | 0.2177 | The interaction between mouse age and genotype is not significantly different | | |
| 9-do (≈ Mid-maturation) | 0.5332 ± 0.1740 (2) | | 0.2398 ± 0.0511(2) |  |  |  |  |  |
| **Tukey’s multiple comparisons test with two-way ANOVA – between age and genotype** | | | | | | | | |
| Groups | p-value | | Groups | p-value | | Groups | p-value | |
| 5-do – WT vs. KI | <0.0001 | *** | WT – 5-do vs. 6-do | 0.9999 | ns | KI – 5-do vs. 6-do | 0.9997 | ns |
| 6-do – WT vs. KI | <0.0001 | *** | WT – 6-do vs. 7-do | >0.999 | ns | KI – 6-do vs. 7-do | >0.999 | ns |
| 7-do – WT vs. KI | <0.0001 | *** | WT – 7-do vs. 8-do | 0.3806 | ns | KI – 7-do vs. 8-do | 0.9953 | ns |
| 8-do – WT vs. KI | 0.0008 | *** | WT – 8-do vs. 9-do | 0.5832 | ns | KI – 8-do vs. 9-do | >0.999 | ns |
| 9-do – WT vs. KI | 0.1020 | ns | WT – 5-do vs. 7-do | 0.9996 | ns | KI – 5-do vs. 7-do | >0.999 | ns |
|  |  |  | WT – 5-do vs. 9-do | 0.0217 | * | KI – 5-do vs. 9-do | 0.9941 | ns |

1. **Supplement Table S11.** RT-qPCR analysis of Ca6 mRNA expression level across the whole enamel organ epithelium layer. The expression level of Ca6 were normalized to β-actin mRNA level. Samples were collected from 12-week-old male and female mice of three genotypes: wild-type (WT), AmelxS16A Knock-in (KI) and heterozygous (HET). Statistical analysis was performed using two-way ANOVA (factors of sex and genotype), followed by multiple post-hoc comparisons.

| ***Ca6* mRNA expression level (ratio to β-actin mRNA) in EOE tissue** | | | | | | | **Two-way ANOVA – between sex and genotype** | | | | |
| --- | --- | --- | --- | --- | --- | --- | --- | --- | --- | --- | --- |
| Sex | | Male | | Female | | | **Variances** | **p-value** | **Significance** | | |
| Genotype | | WT | KI | WT | HET | KI | Sex | 0.7904 | The population means of sexes (m, f) are not significantly different | | |
| Sample 1 | | 0.17143 | 0.09550 | 0.16854 | 0.11538 | 0.06958 |  |  |  |  |  |
| Sample 2 | | 0.17811 | 0.09476 | 0.16922 | 0.09900 | 0.06951 | Genotype | <0.0001 | The population means of genotypes are significantly different | | |
| Sample 3 | | 0.17569 | 0.08046 | 0.18681 | 0.11051 | 0.06159 |  |  |  |  |  |
| Sample 4 | | 0.09458 |  | 0.17821 |  | 0.05206 | Sex * Genotype | 0.0637 | The interaction between sex and genotype is not significantly different | | |
| Mean | | 0.15495 | 0.09024 | 0.17569 | 0.10830 | 0.06319 |  |  |  |  |  |
| SD | | 0.04034 | 0.00848 | 0.00862 | 0.00842 | 0.00831 |  |  |  |  |  |
| **Combined both sexes** | | | | | | | **Tukey’s multiple comparisons test with one-way ANOVA** | | | | |
| Genotype | WT (n=8) | | HET (n=3) | | KI (n=7) | | Groups | | | p-value | |
| Mean±SD | 0.16532 ± 0.02919 | | 0.10830 ± 0.00842 | | 0.07478 ± 0.01636 | | WT vs. KI | | | <0.0001 | *** |
|  | | | | | | | WT vs. HET | | | 0.0055 | ** |
|  |  |  |  |  |  |  | HET vs. KI | | | 0.1148 | ns |

1. **Supplement Table S12.** RT-qPCR analysis of Ca13 mRNA expression level across the whole enamel organ epithelium layer. The expression level of Ca13 were normalized to β-actin mRNA level. Samples were collected from 12-week-old male and female mice of three genotypes: wild-type (WT), AmelxS16A Knock-in (KI) and heterozygous (HET). Statistical analysis was performed using two-way ANOVA (factors of sex and genotype), followed by multiple post-hoc comparisons.

| ***Ca13* mRNA expression level (ratio to β-actin mRNA) in EOE tissue** | | | | | | | **Two-way ANOVA – between sex and genotype** | | | | |
| --- | --- | --- | --- | --- | --- | --- | --- | --- | --- | --- | --- |
| Sex | | Male | | Female | | | **Variances** | **p-value** | **Significance** | | |
| Genotype | | WT | KI | WT | HET | KI | Sex | 0.5914 | The population means of sexes (m, f) are not significantly different | | |
| Sample 1 | | 0.14402 | 0.10924 | 0.14830 | 0.13693 | 0.10729 |  |  |  |  |  |
| Sample 2 | | 0.11313 | 0.11164 | 0.17746 | 0.10964 | 0.09926 | Genotype | 0.0041 | The population means of genotypes are significantly different | | |
| Sample 3 | | 0.12317 | 0.09748 | 0.14258 | 0.13314 | 0.10785 |  |  |  |  |  |
| Sample 4 | | 0.13147 |  | 0.10811 |  | 0.08574 | Sex * Genotype | 0.2477 | The interaction between sex and genotype is not significantly different | | |
| Mean | | 0.12795 | 0.10612 | 0.14411 | 0.12657 | 0.10003 |  |  |  |  |  |
| SD | | 0.01308 | 0.00758 | 0.02845 | 0.01478 | 0.01031 |  |  |  |  |  |
| **Combined both sexes** | | | | | | | **Tukey’s multiple comparisons test with one-way ANOVA** | | | | |
| Genotype | WT (n=8) | | HET (n=3) | | KI (n=7) | | Groups | | | p-value | |
| Mean±SD | 0.13603 ± 0.02225 | | 0.12657 ± 0.01478 | | 0.10264 ± 0.00910 | | WT vs. KI | | | 0.0050 | ** |
|  | | | | | | | WT vs. HET | | | 0.6992 | ns |
|  |  |  |  |  |  |  | HET vs. KI | | | 0.1403 | ns |

1. **Supplement Table S13.** RT-qPCR analysis of Slc4a4 mRNA expression level relative to β-actin mRNA level across five dissected segments of the enamel organ epithelium. Samples were collected from 12-week-old male mice of two genotypes: wild-type (WT) and AmelxS16A Knock-in (KI). Statistical analysis was performed using two-way ANOVA (factors of segment and genotype), followed by multiple post-hoc comparisons.

| ***Slc4a4* mRNA expression level (ratio to β-actin mRNA) in EOE tissue** | | | | | **Two-way ANOVA – between segment and genotype** | | | | |
| --- | --- | --- | --- | --- | --- | --- | --- | --- | --- |
| Genotype  Segment | Male WT (n=4) | | Male KI (n=4) | | **Variances** | **p-value** | **Significance** | | |
|  | Mean | SD | Mean | SD | Segment | <0.0001 | The population means of segments (sec, zam, mat1-3) are significantly different | | |
| Sec | 0.01621 | 0.00420 | 0.00527 | 0.00081 |  |  |  |  |  |
| Zam | 0.02356 | 0.00574 | 0.01712 | 0.00513 | Genotype | 0.0063 | The population means of genotypes (WT and KI) are significantly different | | |
| Mat1 | 0.01858 | 0.00449 | 0.01811 | 0.00434 |  |  |  |  |  |
| Mat2 | 0.01039 | 0.00296 | 0.01220 | 0.00132 | Segment * Genotype | 0.0131 | The interaction between segment and genotype is significantly different | | |
| Mat3 | 0.00841 | 0.00189 | 0.00708 | 0.00308 |  |  |  |  |  |
| **Tukey’s multiple comparisons test with two-way ANOVA – between segment and genotype** | | | | | | | | | |
| Groups | p-value | | Groups | | p-value | | Groups | p-value | |
| Sec – WT vs. KI | 0.0003 | *** | WT – sec vs. zam | | 0.0658 | ns | KI – sec vs. zam | 0.0009 | *** |
| Zam – WT vs. KI | 0.0211 | * | WT – zam vs. mat1 | | 0.3487 | ns | KI – zam vs. mat1 | 0.9956 | ns |
| Mat1 – WT vs. KI | 0.8603 | ns | WT – mat1 vs. mat2 | | 0.0320 | * | KI – mat1 vs. mat2 | 0.1954 | ns |
| Mat2 – WT vs. KI | 0.4993 | ns | WT – mat2 vs. mat3 | | 0.0049 | ** | KI – mat2 vs. mat3 | 0.3219 | ns |
| Mat3 – WT vs. KI | 0.6190 | ns | WT – sec vs. mat1 | | 0.8963 | ns | KI – sec vs. mat1 | 0.0003 | *** |
|  |  |  | WT – zam vs. mat3 | | <0.0001 | *** | KI – zam vs. mat3 | 0.0056 | ** |

1. **Supplement Table S14.** RT-qPCR analysis of Slc24a4 mRNA expression level relative to β-actin mRNA level across five dissected segments of the enamel organ epithelium. Samples were collected from 12-week-old male mice of two genotypes: wild-type (WT) and AmelxS16A Knock-in (KI). Statistical analysis was performed using two-way ANOVA (factors of segment and genotype), followed by multiple post-hoc comparisons.

| ***Slc24a4* mRNA expression level (ratio to β-actin mRNA) in EOE tissue** | | | | | **Two-way ANOVA – between segment and genotype** | | | | |
| --- | --- | --- | --- | --- | --- | --- | --- | --- | --- |
| Genotype  Segment | Male WT (n=3) | | Male KI (n=3) | | **Variances** | **p-value** | **Significance** | | |
|  | Mean | SD | Mean | SD | Segment | <0.0001 | The population means of segments (sec, zam, mat1-3) are significantly different | | |
| Sec | 3.68750 | 0.44474 | 0.22935 | 0.05590 |  |  |  |  |  |
| Zam | 14.15627 | 1.92452 | 5.41339 | 0.47187 | Genotype | <0.0001 | The population means of genotypes (WT and KI) are significantly different | | |
| Mat1 | 8.07931 | 1.13295 | 7.01271 | 1.05221 |  |  |  |  |  |
| Mat2 | 1.41124 | 0.42934 | 2.27426 | 0.14499 | Segment * Genotype | <0.0001 | The interaction between segment and genotype is significantly different | | |
| Mat3 | 0.47207 | 0.12751 | 0.60991 | 0.13087 |  |  |  |  |  |
| **Tukey’s multiple comparisons test with two-way ANOVA – between segment and genotype** | | | | | | | | | |
| Groups | p-value | | Groups | | p-value | | Groups | p-value | |
| Sec – WT vs. KI | <0.0001 | *** | WT – sec vs. zam | | <0.0001 | *** | KI – sec vs. zam | <0.0001 | *** |
| Zam – WT vs. KI | <0.0001 | *** | WT – zam vs. mat1 | | <0.0001 | *** | KI – zam vs. mat1 | 0.2214 | ns |
| Mat1 – WT vs. KI | 0.1333 | ns | WT – mat1 vs. mat2 | | <0.0001 | *** | KI – mat1 vs. mat2 | <0.0001 | *** |
| Mat2 – WT vs. KI | 0.2508 | ns | WT – mat2 vs. mat3 | | 0.6517 | ns | KI – mat2 vs. mat3 | 0.1901 | ns |
| Mat3 – WT vs. KI | 0.8424 | ns | WT – sec vs. mat1 | | <0.0001 | *** | KI – sec vs. mat1 | <0.0001 | *** |
|  |  |  | WT – zam vs. mat3 | | <0.0001 | *** | KI – zam vs. mat3 | <0.0001 | *** |

1. **Supplement Table S15.** RT-qPCR analysis of Slc24a3 mRNA expression level relative to β-actin mRNA level across five dissected segments of the enamel organ epithelium. Samples were collected from 12-week-old male mice of two genotypes: wild-type (WT) and AmelxS16A Knock-in (KI). Statistical analysis was performed using two-way ANOVA (factors of segment and genotype), followed by multiple post-hoc comparisons.

| ***Slc24a3* mRNA expression level (ratio to β-actin mRNA) in EOE tissue** | | | | | **Two-way ANOVA – between segment and genotype** | | | | |
| --- | --- | --- | --- | --- | --- | --- | --- | --- | --- |
| Genotype  Segment | Male WT (n=4) | | Male KI (n=4) | | **Variances** | **p-value** | **Significance** | | |
|  | Mean | SD | Mean | SD | Segment | <0.0001 | The population means of segments (sec, zam, mat1-3) are significantly different | | |
| Sec | 0.11959 | 0.01873 | 0.11322 | 0.02052 |  |  |  |  |  |
| Zam | 0.03633 | 0.01001 | 0.12363 | 0.02046 | Genotype | 0.0001 | The population means of genotypes (WT and KI) are significantly different | | |
| Mat1 | 0.01104 | 0.00118 | 0.01404 | 0.00140 |  |  |  |  |  |
| Mat2 | 0.00870 | 0.00119 | 0.00756 | 0.00073 | Segment * Genotype | <0.0001 | The interaction between segment and genotype is significantly different | | |
| Mat3 | 0.00922 | 0.00226 | 0.00610 | 0.00132 |  |  |  |  |  |
| **Tukey’s multiple comparisons test with two-way ANOVA – between segment and genotype** | | | | | | | | | |
| Groups | p-value | | Groups | | p-value | | Groups | p-value | |
| Sec – WT vs. KI | 0.4362 | ns | WT – sec vs. zam | | <0.0001 | *** | KI – sec vs. zam | 0.6993 | ns |
| Zam – WT vs. KI | <0.0001 | *** | WT – zam vs. mat1 | | 0.0292 | * | KI – zam vs. mat1 | <0.0001 | *** |
| Mat1 – WT vs. KI | 0.7127 | ns | WT – mat1 vs. mat2 | | 0.9984 | ns | KI – mat1 vs. mat2 | 0.9277 | ns |
| Mat2 – WT vs. KI | 0.8886 | ns | WT – mat2 vs. mat3 | | >0.9999 | ns | KI – mat2 vs. mat3 | 0.9997 | ns |
| Mat3 – WT vs. KI | 0.7018 | ns | WT – sec vs. mat1 | | <0.0001 | *** | KI – sec vs. mat1 | <0.0001 | *** |
|  |  |  | WT – zam vs. mat3 | | 0.0169 | * | KI – zam vs. mat3 | <0.0001 | *** |

1. **Supplement Table S16.** RT-qPCR analysis of Cftr mRNA expression level relative to β-actin mRNA level across five dissected segments of the enamel organ epithelium. Samples were collected from 12-week-old male mice of two genotypes: wild-type (WT) and AmelxS16A Knock-in (KI). Statistical analysis was performed using two-way ANOVA (factors of segment and genotype), followed by multiple post-hoc comparisons.

| ***Cftr* mRNA expression level (ratio to β-actin mRNA) in EOE tissue** | | | | | **Two-way ANOVA – between segment and genotype** | | | | |
| --- | --- | --- | --- | --- | --- | --- | --- | --- | --- |
| Genotype  Segment | Male WT (n=3) | | Male KI (n=3) | | **Variances** | **p-value** | **Significance** | | |
|  | Mean | SD | Mean | SD | Segment | <0.0001 | The population means of segments (sec, zam, mat1-3) are significantly different | | |
| Sec | 1.05564 | 1.00723 | 0.10220 | 0.16148 |  |  |  |  |  |
| Zam | 4.27808 | 0.57732 | 2.85731 | 0.75994 | Genotype | <0.0001 | The population means of genotypes (WT and KI) are significantly different | | |
| Mat1 | 5.01410 | 0.59268 | 2.52402 | 0.75434 |  |  |  |  |  |
| Mat2 | 5.68130 | 0.91846 | 2.06227 | 0.94251 | Segment * Genotype | 0.0110 | The interaction between segment and genotype is significantly different | | |
| Mat3 | 5.24066 | 0.46263 | 1.63234 | 0.61447 |  |  |  |  |  |
| **Tukey’s multiple comparisons test with two-way ANOVA – between segment and genotype** | | | | | | | | | |
| Groups | p-value | | Groups | | p-value | | Groups | p-value | |
| Sec – WT vs. KI | 0.1209 | ns | WT – sec vs. zam | | 0.0002 | *** | KI – sec vs. zam | 0.0012 | ** |
| Zam – WT vs. KI | 0.0255 | * | WT – zam vs. mat1 | | 0.7228 | ns | KI – zam vs. mat1 | 0.9785 | ns |
| Mat1 – WT vs. KI | 0.0004 | *** | WT – mat1 vs. mat2 | | 0.7871 | ns | KI – mat1 vs. mat2 | 0.9321 | ns |
| Mat2 – WT vs. KI | <0.0001 | *** | WT – mat2 vs. mat3 | | 0.9421 | ns | KI – mat2 vs. mat3 | 0.9468 | ns |
| Mat3 – WT vs. KI | <0.0001 | *** | WT – sec vs. mat1 | | <0.0001 | *** | KI – sec vs. mat1 | 0.0043 | ** |
|  |  |  | WT – zam vs. mat3 | | 0.4933 | ns | KI – zam vs. mat3 | 0.2662 | ns |
